# Supplementary material for: Knowledge and preventive barriers towards conducting systematic review among undergraduate medical students of Arab countries: A multi country online survey
Source: PLoS One. 2025 Aug 18;20(8):e0329827. doi: 10.1371/journal.pone.0329827 (PMC12360549; doi:10.1371/journal.pone.0329827)
Supplement: S1 Table — (DOCX) [file pone.0329827.s001.docx]

| **Supplementary table 1:** Level of knowledge and practice of systematic review according to countries. | | | | | | | | | | | | |
| --- | --- | --- | --- | --- | --- | --- | --- | --- | --- | --- | --- | --- |
| **Variables** | **N** | **Overall**, N = 13,060*^1^* | **Country** | | | | | | | | | **p-value***^2^* |
|  |  |  | **Algeria**, N = 725*^1^* | **Egypt**, N = 2,202*^1^* | **Jordan**, N = 851*^1^* | **Libya**, N = 1,023*^1^* | **Palestine**, N = 586*^1^* | **Saudi Arabia**, N = 2,160*^1^* | **Sudan**, N = 4,195*^1^* | **Syria**, N = 905*^1^* | **Yemen**, N = 413*^1^* |  |
| **Level of knowledge about systematic reviews** | 13,060 |  |  |  |  |  |  |  |  |  |  | **<0.001** |
| High |  | 563 (4.3%) | 9 (1.2%) | 118 (5.4%) | 30 (3.5%) | 6 (0.6%) | 31 (5.3%) | 233 (10.8%) | 94 (2.2%) | 31 (3.4%) | 11 (2.7%) |  |
| Low |  | 12,497 (95.7%) | 716 (98.8%) | 2,084 (94.6%) | 821 (96.5%) | 1,017 (99.4%) | 555 (94.7%) | 1,927 (89.2%) | 4,101 (97.8%) | 874 (96.6%) | 402 (97.3%) |  |
| **Have you ever enrolled in or participated in a systematic review? (Yes)** | 13,060 | 1,567 (12.0%) | 40 (5.5%) | 392 (17.8%) | 105 (12.3%) | 93 (9.1%) | 65 (11.1%) | 296 (13.7%) | 435 (10.4%) | 43 (4.8%) | 98 (23.7%) | **<0.001** |
