# Supplementary material for: Knowledge and preventive barriers towards conducting systematic review among undergraduate medical students of Arab countries: A multi country online survey
Source: PLoS One. 2025 Aug 18;20(8):e0329827. doi: 10.1371/journal.pone.0329827 (PMC12360549; doi:10.1371/journal.pone.0329827)
Supplement: Attachment — Submitted filename: PONE-D-23-31908_reviewer reviewed by DA.pdf [file pone.0329827.s004.pdf]

PLOS ONE

Knowledge, practice, and perceived barriers towards conducting systematic reviews in undergraduate medical students of Arab countries: A multi-country online surve

--Manuscript Draft--

**Manuscript Number:**	PONE-D-23-31908
**Article Type:**	Research Article
**Full Title:**	Knowledge, practice, and perceived barriers towards conducting systematic reviews in undergraduate medical students of Arab countries: A multi-country online surve
**Short Title:**	Knowledge; Practice; Perceived barriers; Systematic reviews; Medical students
**Corresponding Author:**	Elfatih A. Hasabo<br>University of Khartoum Faculty of Medicine<br>Khartoum, SUDAN
**Keywords:**	knowledge; practice; Perceived barriers; systematic reviews; Medical Students
**Abstract:**	Background: Systematic reviews are the highest level of evidence. Medical students are encouraged to learn how to conduct systematic reviews. Barriers to conducting systematic reviews should be known to help increase the Practice of systematic reviews. We aimed to assess knowledge, Practice and perceived barriers to conducting systematic reviews of undergraduate medical students of Arabs countries.<br>Methods: A cross-sectional study was conducted on undergraduate medical students from nine Arab countries studying at public or private medical schools. Sociodemographic Information, knowledge, Practice, and barriers to conducting systematic reviews were obtained from participants via an online survey. The level of knowledge about systematic reviews was measured using several questions with a total score of 19 (More than nine is good knowledge, and nine or less is poor knowledge).<br>Results: A total of 13060 participants from nine Arab countries participated. Most of them were females (58.9%), and 77.0% were studying in a public university. Also, 49.0% of them were in clinical years. Only 31% heard about systematic reviews, and 3,275 (25.1%) participants attended or received training on systematic reviews. The overall good knowledge of systematic reviews was 4.3%. The most identified barriers to conducting systematic reviews were a lack of knowledge about systematic review (47.0%), followed by a lack of research exposure and opportunities (28.8%). Regarding Practice, only 1567 (12.0%) participants participated or enrolled in a systematic review research project. Only 471/1567 (30.1%) published secondary research. The types of enrolled research projects were systematic reviews (62.3%), systematic review and meta-analysis (43.3%), and network meta-analysis (33.4%).<br>Conclusion: There was a low level of knowledge and Practice of systematic review among participants. Also, several barriers prevent undergraduate medical students from conducting systematic reviews. Further training about systematic reviews should be established to increase the knowledge of systematic reviews in undergraduate medical students.
**Order of Authors:**	Elfatih A. Hasabo, MBBS
	Walaa Elnaiem
	Alaa S. Ahmed
	Azza A. Abbas, MBBS
	MOHAMED SATI SHAMPOOL, MBBS
	Khabab Abbasher Hussein Mohamed Ahmed, MBBS
	Ghassan Elfatih Mustafa Ahmed, MBBS
	Mohammed Mahmmoud Fadelallah Eljack, MBBS
	Alamin Alfatih, MBBS
	Huda A Sherif

Powered by Editorial Manager® and ProduXion Manager® from Aries Systems Corporation

	Omer Al Komi
	Hajar aldare
	Amira Yasmine Benmelouka
	Afnan W. M. Jobran
	Tayba Mugibel
	MOHAMAD IMAD AL-KASSIH
	Muhamad Zakaria Brimo Alsaman, M.D.
	Ahmed Aljabali
**Additional Information:**	
**Question**	**Response**
**Financial Disclosure**<br><br>Enter a financial disclosure statement that describes the sources of funding for the work included in this submission. Review the submission guidelines for detailed requirements. View published research articles from *PLOS ONE* for specific examples.<br><br>This statement is required for submission and **will appear in the published article** if the submission is accepted. Please make sure it is accurate.<br><br>**Funded studies**<br>Enter a statement with the following details:<br>• Initials of the authors who received each award<br>• Grant numbers awarded to each author<br>• The full name of each funder<br>• URL of each funder website<br>• Did the sponsors or funders play any role in the study design, data collection and analysis, decision to publish, or preparation of the manuscript?<br><br>Did you receive funding for this work?	The author(s) received no specific funding for this work.
**Competing Interests**<br><br>Use the instructions below to enter a competing interest statement for this submission. On behalf of all authors, disclose any competing interests that could be perceived to bias this	The authors have declared that no competing interests exist.

work—acknowledging all financial support and any other relevant financial or non-financial competing interests.<br><br>This statement is required for submission and **will appear in the published article** if the submission is accepted. Please make sure it is accurate and that any funding sources listed in your Funding Information later in the submission form are also declared in your Financial Disclosure statement.<br><br>View published research articles from *PLOS ONE* for specific examples.<br><br>**NO authors have competing interests**<br><br>Enter: *The authors have declared that no competing interests exist.*<br><br>**Authors with competing interests**<br><br>Enter competing interest details beginning with this statement:<br><br>*I have read the journal's policy and the authors of this manuscript have the following competing interests: [insert competing interests here]*<br><br>* typeset	
**Ethics Statement**<br><br>Enter an ethics statement for this submission. This statement is required if the study involved:<br><br>• Human participants<br>• Human specimens or tissue<br>• Vertebrate animals or cephalopods<br>• Vertebrate embryos or tissues<br>• Field research<br><br>Write "N/A" if the submission does not require an ethics statement.	Ethical approval of this study (Approval no: 4-22, 1/2/2022) was obtained from the Health-Sector Ethical Review Committee in the Faculty of Medicine, University of Gezira, Gezira, Sudan. The study was carried out following the relevant ethical guidelines and regulations. The participants were asked to give consent that they agree to participate in the study by filling the questionnaire for research purposes in the online form, and all the participants provided informed written consent after providing a clear explanation of the study purpose.

Powered by Editorial Manager® and ProduXion Manager® from Aries Systems Corporation

General guidance is provided below. Consult the submission guidelines for detailed instructions. **Make sure that all information entered here is included in the Methods section of the manuscript.**<br><br>**Format for specific study types**<br><br>**Human Subject Research (involving human participants and/or tissue)**<br>• Give the name of the institutional review board or ethics committee that approved the study<br>• Include the approval number and/or a statement indicating approval of this research<br>• Indicate the form of consent obtained (written/oral) or the reason that consent was not obtained (e.g. the data were analyzed anonymously)<br><br>**Animal Research (involving vertebrate animals, embryos or tissues)**<br>• Provide the name of the Institutional Animal Care and Use Committee (IACUC) or other relevant ethics board that reviewed the study protocol, and indicate whether they approved this research or granted a formal waiver of ethical approval<br>• Include an approval number if one was obtained<br>• If the study involved *non-human primates*, add *additional details* about animal welfare and steps taken to ameliorate suffering<br>• If anesthesia, euthanasia, or any kind of animal sacrifice is part of the study, include briefly which substances and/or methods were applied<br><br>**Field Research**<br><br>Include the following details if this study involves the collection of plant, animal, or other materials from a natural setting:<br>• Field permit number<br>• Name of the institution or relevant body that granted permission	
**Data Availability**<br><br>Authors are required to make all data underlying the findings described fully	No - some restrictions will apply

available, without restriction, and from the time of publication. PLOS allows rare exceptions to address legal and ethical concerns. See the PLOS Data Policy and FAQ for detailed information.<br><br>A Data Availability Statement describing where the data can be found is required at submission. Your answers to this question constitute the Data Availability Statement and **will be published in the article**, if accepted.<br><br>**Important:** Stating 'data available on request from the author' is not sufficient. If your data are only available upon request, select 'No' for the first question and explain your exceptional situation in the text box.<br><br>Do the authors confirm that all data underlying the findings described in their manuscript are fully available without restriction?	
**Describe where the data may be found in full sentences. If you are copying our sample text, replace any instances of XXX with the appropriate details.**<br><br>• If the data are **held or will be held in a public repository**, include URLs, accession numbers or DOIs. If this information will only be available after acceptance, indicate this by ticking the box below. For example: *All XXX files are available from the XXX database (accession number(s) XXX, XXX.*).<br>• If the data are all contained **within the manuscript and/or Supporting Information files**, enter the following: *All relevant data are within the manuscript and its Supporting Information files.*<br>• If neither of these applies but you are able to provide **details of access elsewhere**, with or without limitations, please do so. For example:<br><br>*Data cannot be shared publicly because of [XXX]. Data are available from the XXX Institutional Data Access / Ethics*	The datasets generated and/or analyzed during the current study are not publicly available due to privacy but are available from the corresponding author on reasonable request.

Committee (contact via XXX) for researchers who meet the criteria for access to confidential data.<br><br>*The data underlying the results presented in the study are available from (include the name of the third party and contact information or URL).*<br>• This text is appropriate if the data are owned by a third party and authors do not have permission to share the data.<br><br>* typeset	
Additional data availability information:	

Powered by Editorial Manager® and ProduXion Manager® from Aries Systems Corporation

Manuscript

Click here to access/download;Manuscript;Final manuscript edited (16.11.2022)plos.docx

Knowledge, practice, and perceived barriers towards conducting systematic reviews in undergraduate medical students of Arab countries: A multi-country online survey

Elfatih A. Hasabo[1, 2#*], Walaa Elnaiem[1,2#], Alaa S. Ahmed[1,2], Azza A. Abbas[1,2], Khabab Abbasher Hussien Mohamed Ahmed[1,2], Ghassan Elfatih Mustafa Ahmed[1,2], MOHAMED SATI SHAMPOOL[1,2], Mohammed Mahmmoud Fadelallah Eljack[2,3], Alamin Alfatih[1,2], Huda A Sherif[2,4], Omar Al Komi[2,5], Hajar aldare[2,6], Amira Yasmine Benmelouka[2,7], Afnan W. M. Jobran[2,8], Tayba Mugibel[2,9], MOHAMAD IMAD AL-KASSIH[2,5], Muhamad Zakaria Brimo Alsaman [2,10], Ahmed Aljabali[2,11], Sudan Analytics Research Group team of collaborators[12]

Affiliation:

1. Faculty of Medicine, University of Khartoum, Khartoum, Sudan
2. Sudan Analytics Research Group, Khartoum, Sudan
3. Faculty of Medicine, University of Bakht Alruda, Ed Dueim, Sudan
4. Faculty of Medicine, University of Menofia, Menofia, Egypt
5. College of Medicine, Sulaiman Alrajhi University, Albukayriyah, Al-Qassim, Saudi Arabia
6. Faculty of Medicine, University of Sabha, Sabha, Libya
7. Faculty of Medicine, University of Algiers, Algiers, Algeria
8. Faculty of Medicine, Al Quds University, Jerusalem, Palestine, State of
9. College of Medicine, Hadhramout University, Hadhramout, Yemen
10. Vascular surgery Department, Al-Razi Hospital, Aleppo, Syria.
11. Faculty of Medicine, Jordan University of Science and Technology, Irbid, Jordon.
12. Collaborators are mentioned in the Acknowledgement section.

[#]These author has an equal contribution, and both were the first author

***Corresponding author**: Faculty of Medicine, University of Khartoum, ElQasr Avenue, Khartoum, Khartoum state, 11111, Sudan.

Email: elfatih.ahmed.hasabo@gmail.com

ORCID: https://orcid.org/0000-0001-9727-8620

Authors and ORCID

1. Elfatih A. Hasabo, Faculty of Medicine, University of Khartoum, Khartoum, Sudan. elfatih.ahmed.hasabo@gmail.com, ORCID: 0000-0001-9727-8620
2. Walaa Elnaiem, Faculty of Medicine, University of Khartoum, Khartoum, Sudan. Walaaabdalgadir@gmail.com, ORCID: 0000-0002-4852-4730
3. Alaa S. Ahmed, Faculty of Medicine, University of Khartoum, Khartoum, Sudan. alaa.salah23699@gmail.com
4. Azza A. Abbas, Faculty of Medicine, University of Khartoum, Khartoum, Sudan. Azzaalfadil8@gmail.com
5. Khabab Abbasher Hussien Mohamed Ahmed, Faculty of Medicine, University of Khartoum, Khartoum, Sudan.Khabab9722@gmail.com, ORCID: 0000-0003-4608-5321
6. Ghassan Elfatih Mustafa Ahmed, Faculty of Medicine, University of Khartoum, Khartoum, Sudan. nimirghassan@gmail.com, ORCID: 0000-0002-0258-6282
7. MOHAMED SATI SHAMPOOL, Faculty of Medicine, University of Khartoum, Khartoum, Sudan. mohamedsati@yahoo.com
8. Mohammed Mahmmoud Fadelallah Eljack, Faculty of Medicine, University of Bakht Alruda, Eldueim, Sudan. m.mahmmoud96@gmail.com, ORCID: 0000-0002-2370-9368
9. Alamin Alfatih, Faculty of medicine, University of Khartoum, Khartoum, Sudan. Elaminelfatih4444@gmail.com
10. Huda A Sherif, Faculty of Medicine, University of Menofia, Menoufia, Egypt. Hudasherif677@gmail.com
11. Omar Al Komi, College of Medicine, Sulaiman Alrajhi University, Albukayriyah, Al-Qassim, Saudi Arabia.omar.alkomi@outlook.com, ORCID: 0000-0001-8537-2521
12. Hajar aldare, Faculty of Medicine, University of Sabha, Libya. Doctorhajar1@gmail.com
13. Amira Yasmine Benmelouka, Faculty of Medicine, University of Algiers, Algiers, Algeria. amira.yasmine.benmelouka@gmail.com, ORCID: 0000-0001-7618-4908
14. Afnan W. M. Jobran, Faculty of Medicine, Al Quds University, Jerusalem, Palestine. afnanjobran26@gmail.com, ORCID: 0000-0003-1068-2984
15. Tayba Mugibel, Hadhramout University College of Medicine. taibamog114@gmail.com, ORCID: 0000-0001-5220-3174

16. MOHAMAD IMAD AL-KASSIH, STUDENT, Suliman Alrjhi University, Saudi Arabia. MEK9924@GMAIL.COM
17. Muhamad Zakaria Brimo Alsaman, M.D, Vascular Surgery Department, Al-Razi Hospital, Aleppo, Syria. dr.zakariaBrimo@gmail.com, ORCID: 0000-0003-4453-4699
18. Ahmed Aljabali, Faculty of Medicine, Jordan University of Science and Technology, Irbid, Jordan. aljbalia37@gmail.com

Sudan Analytics Research Group team of collaborators: Abdelrahim A Ahmed, Abdelrhman mohamed abdelrady, Abdelrhman Muwafaq Janem, Abdelrhman Waleed Elshenawy, Abdulghani Arja, Abdullah alshogaa, Abdullah Nasser Ali Hamilah, Abdurahim Mustafa Alaswad, Abeer S. El-Shawady, Abubakr Muhammad, Afaf Ahmad, Afnan Mousa Alhawsawi, Ahmad Ali Essa, Ahmed Khalid Yousif Mustafa, Al Zahraa Ali, Alaa Amr, Albushra A. Adam, Ali Alkhamis, Amna Altayeb Alhaj Ahmed, Aml Mostafa Farid, Amr Mahmoud Abdelraoof, Asma Ali alarabi, Asmaa H Alzabadiah, Awab Osman Ahmed Osman, Aya Ahmed Mohamed, Aya.M.abbas, Bashar Bazkke, DarElslam ELawad Osman, Deena faraj abdulali altarjami, Douaa Effat Belal, Ebtihal Mohammed Albasheir, Elsayed Hammad, Enaam alabed ahmed adam, Entisar Abdalla Zin Elabdein Ahmed, Esra Magzoub Ibrahem Ahmed, Esraa Salaheldin Ahmed Alfadul, Essraa ali alhudhairy, Faris Hamed, Ghadeer Ahmed Alhawsawi, Hagar K Elgazar, Haifa saif aldeen Alsaid humid, Haneen A. Banihani, Hassan Elamin Hassan Elamin, Hawa Essa Mohammed, Haya Deeb, Heba Alwaa, Hosam Khalouf, Hussein Mohammed Hussein Ghalib, ihcene Telaidji, Intisar Bubaker Owhida, Ismail Adam, Khadija Tariq Habib, Khaled Albakri, Khaled Tawfeeq, Khalid Elsir Idris, Khawla Alshahrani, Khetam Mohamed Saleh Adah, Khulod Ali Bokir, Kinda ahmed hardan, Lubna Hossam Aloufi, Lulyah almallah, Maazouz Bensalem, Maisa Nazzal Ayman Nazzal, Majid H Ali, Makhlouf Sofia, Manar Kahoul, Mawahib Elsheikh, Mawda Jumma, Mazin Mohammed Osman, Moath Almekhlafi, Moath Rushdi Atiani, Mobtahel Modather mohammed omer, Moftah Imhamed Moftah Himeda, Mohamed Abdelmonem, Mohamed Baklola, Mohamed Baraka, Mohamed Faroug Ali Yassin, Mohamed Gamal Eldow Abbas, Mohamed Ibrahem Baklola, Mohammad Abdulrahman Aljundi, Mohammad Saleh Alwajih, Mohammed Amir Rais, Mohmmad awd alsyed ahmad abbass, Monzer Elgamal, Malaz Omer Altegani Mohammed, Muhammad Sayed Muhammad Masoud, Mutwakel Ali Mohammed, Nagwa yassin Mohammad Taha, Nisreen Abufaris, Noon Isam Eldin Hasssan Hagmusa,

Noura Ghazi Abu Lehia, Nourhan Elsamahy, Omar Abdulraheem Baqais, Omar Riffi, Omar S. Aldhafeeri, Omer A. Idrees, Qosay Sami Jabr, Raghad Nabeel Sadi Saleh, Rahaf mutlaq alotaibi, Rasha Nasser Hasnah, Rawan AbdAlwahd Mohammed Osman, Reema Saleh Zayed Alamri, Riham Mohammed Osman Mohammed, Roaa B. Albashir, Roua Arian, Ruwayda Abdulmutalib mawloud, Selema Nabeel Belgasem, Shahd T. Idais, Somayya Tabsho, Thekra Fawaz Albeshri, Turfa Moudarres, Waad Abu Elbeada, Wafaa Eshag Moheyeldeen, Yousef Abdullah Aldreweesh, Ziad Alahmad, Rawa Naimat, Majedah A. Alakayleh, Shahed N. Abughanam, Mallak I. Alja'fari, Razan M. Altarawaneh, Ihab A. Alsalamin, Duoaa Albelal, Ahmad Aissa.

Abstract

Background: Systematic reviews are the highest level of evidence. Medical students are encouraged to learn how to conduct systematic reviews. Barriers to conducting systematic reviews should be known to help increase the Practice of systematic reviews. We aimed to assess knowledge, Practice and perceived barriers to conducting systematic reviews of undergraduate medical students of Arabs countries.

Methods: A cross-sectional study was conducted on undergraduate medical students from nine Arab countries studying at public or private medical schools. Sociodemographic Information, knowledge, Practice, and barriers to conducting systematic reviews were obtained from participants via an online survey. The level of knowledge about systematic reviews was measured using several questions with a total score of 19 (More than nine is good knowledge, and nine or less is poor knowledge).

Results: A total of 13060 participants from nine Arab countries participated. Most of them were females (58.9%), and 77.0% were studying in a public university. Also, 49.0% of them were in clinical years. Only 31% heard about systematic reviews, and 3,275 (25.1%) participants attended or received training on systematic reviews. The overall good knowledge of systematic reviews was 4.3%. The most identified barriers to conducting systematic reviews were a lack of knowledge about systematic review (47.0%), followed by a lack of research exposure and opportunities (28.8%). Regarding Practice, only 1567 (12.0%) participants participated or enrolled in a systematic review research project. Only 471/1567 (30.1%) published secondary research. The types of enrolled research projects were systematic reviews (62.3%), systematic review and meta-analysis (43.3%), and network meta-analysis (33.4%).

Conclusion: There was a low level of knowledge and Practice of systematic review among participants. Also, several barriers prevent undergraduate medical students from conducting systematic reviews. Further training about systematic reviews should be established to increase the knowledge of systematic reviews in undergraduate medical students.

Keywords: Knowledge; Practice; Perceived barriers; Systematic review; Medical students

Introduction

A systematic literature review (SLR) is a qualitative synthesis of information on a specific research question; it is carried out in accordance with a prespecified protocol to objectively select the articles that will be included in our study. It was first used in 1990 as part of evidence-based medicine (EBM) in response to growing research-to-practice gaps. It has since become one of the most popular types of research review, with over 15,000 publications labelled as systematic reviews in PubMed in 2018 [123]. A systematic literature review (SLR) can result in a meta-analysis (MA) in some circumstances if sufficient data allows us to utilize statistical tools to produce a quantitative synthesis of the information gathered from the literature. When conducting a meta-analysis, it is essential to remember that it is a statistical procedure representing the pooled effect of all of the studies included in the systematic literature review to provide an overall result; as such, it is an objective evaluation of the information based on numerical data [4]. Different purposes can be served by systematic literature reviews, including keeping physicians up to date on a specific topic, evaluating the methodological quality of published studies, identifying knowledge gaps when no new research has been published, and addressing knowledge gaps when there are discrepancies in the literature, amongst others [5].

Modern clinical research considers systemic literature reviews of clinical trials as the best evidence. If done correctly, systemic literature reviews of clinical trials can save resources by preventing the development of unnecessary clinical trials [6]. However, to conduct a systemic literature review, researchers must first understand its approach, which can be difficult, particularly in developing countries where research mentoring is lacking [7]. Using systematic reviews, researchers want to uncover all relevant individual studies on a particular health-related topic and synthesize their conclusions, making the available information more accessible to decision-makers [4].

Even though it is thought to present a proper method for assessing the relative effectiveness of different interventions that seek to implement research evidence into healthcare practice, literature has recently emerged that offers contradictory findings about systematic reviews that may be misleading. This could be due to a lack of a well-known or consensus definition of secondary studies that analyze them or because the definition is too broad [82].

There are different barriers facing the uptake of systematic review, including financial problems, the sheer volume of primary research, the difficulties in applying global evidence

from a local clinical context, limited time and impaired awareness of evidence sources, limited critical appraisal skills, and the limited relevance of research findings [9].

In general, there was a significant gap in research in Arab countries, and even when it was identified, it was frequently of poor quality. This might be remedied by including medical students in research projects, starting with research assistantships to gain experience and develop scientific thinking skills in the short term, and subsequently as physician-scientists to meet the growing demand for physician-scientists in the long run [10].

Because systematic reviews are a common type of research and evidence-based medicine, they should be included in medical school curricula to help students learn about literature searches, data collection, and analysis, as well as research knowledge and skills, a better understanding of scientific findings, and career goals, all of which are aligned with the goals of the World Federation of Medical Education [11].

To date, there have been no attempts to examine systematic review practices in middle east countries. Also, no data scientifically examined the knowledge and Practice of systematic review among medical students worldwide, necessitating this study to describe Awareness, Practice, and perceived barriers of systematic reviews among undergraduate medical students. Furthermore, it is to determine which demographic characteristics are more associated with good knowledge and less perceived barriers.

Methods

Study design and settings

A cross-sectional was conducted via online survey among undergraduate medical students who were studying in private or public universities in nine Arab countries (Algeria, Egypt, Jordan, Libya, Palestine, Saudi Arabia, Sudan, Syria, and Yemen) from 3rd of January 2022 till 26th of June 2022 (the recruitment period) to measure the level of knowledge, Practice, and perceived barriers towards conducting systematic reviews.

The online survey was distributed to all selected universities by collaborators in included Arabs countries among undergraduate students. The number of included participants from each country and map are shown in Figure 1.

Participants:

We included all undergraduate medical students older than 18 years studying at the Faculty of Medicine in included universities of selected Arabs contraries who were eligible to participate in our online survey. All graduate students and students not studying at the faculty of medicine were excluded from the study.

Instruments used to measure the variables of interest in the study:

Our questionnaire was designed after an extensive literature review and converted into an online Google form to collect the following information from participants:

- Socioeconomic and characteristic information about participants includes age, gender, residence, marital status, type of university, year of study, having a family member in the healthcare field, and participating in the research-related activity.
- The level of knowledge about systematic review consists of 19 questions to measure the level of knowledge among participants - the total knowledge score was 19-. Participants have to answer these questions. If they answered more than nine questions correctly, they would be considered good knowledge, and if they answered nine questions or less correctly, they will be considered poor.
- Awareness about Medical databases: Participants were listed for several medical databases and asked to identify databases they knew.
- Rating skills of the systematic review were done using 5-point Likert scale answers in 5 scales from poor {1} to advanced {5} for 11 steps of conducting a systematic review.

- Barriers to conducting systematic reviews: participants were asked to identify the barriers that prevent them from conducting systematic reviews.
- Lastly, the Practice of systematic reviews is assessed via several questions asked of participants.

Before distributing the questionnaire to participants, we checked face to content validity and relevance of the questions. Also, pilot testing was done to ensure the clarity of the questions of the questionnaire.

Data collection and Sampling:

Collaborators from included countries were recruited using google Forms after posting an announcement for recruitment by Sudan Analytics account on social media (Telegram, Facebook, LinkedIn,...etc.). Convenient sampling was used in this study to include every participant who wanted to enrol in our online survey due to the inability to conduct a random sampling due to the COVID -19 pandemic and a large number of included countries and universities. Collaborators distributed the online Google form for participants in their universities, and they sent regular reminders during the study period for undergraduate medical students to enrol in our study.

Sample size calculation:

After using a 95% Confidence Interval (CI), 50% response distribution, and 0.05 margin of error in the following formula $n = z^2P(1-P)/d^2$, a total number of 384 participants from each selected country was required to represent each country.

Ethics approval and consent to participate:

Ethical approval of this study (Approval no: 4-22, 1/2/2022) was obtained from the Health-Sector Ethical Review Committee in the Faculty of Medicine, University of Gezira, Gezira, Sudan. The study was carried out following the relevant ethical guidelines and regulations. The participants were asked to give consent that they agree to participate in the study by filling the questionnaire for research purposes in the online form, and all the participants provided informed written consent after providing a clear explanation of the study purpose.

Statistical analysis:

We presented data as mean and standard deviation (S.D) for continuous variables and percentages for categorical variables. Data were categorized according to the level of

knowledge about systematic review and enrolment of participants in secondary research. Test of significant difference – Chi-square, Fisher exact test, and independent T-test- were used to find if there were significant differences in variables of our study between participants with good versus poor knowledge about systematic review and between participants who enrolled or didn't enrol in secondary research. R software version 4.0.2 was used in analysis.

Required to address the questions:

1. What was the response rate
2. How to manage missing cases because as being online survey it was expected to have missing cases
3. This was multi country study how to ensure data safety and security

Suggested to add another two section
1. Data management and
2. Survey administration

Results

Participants characteristics

A total of 13060 medical students participated in this study from, nine different Arab countries. Most of the students were from Sudan (32.1%), followed by Egypt (16.9%), Saudi Arabia (16.5%), Libya (7.8%), Syria (6.9%), Jordan (6.5%), Algeria (5.6%), Palestine (4.5%), and Yemen (3.2%), (Figure 1). The mean age of the students was 21.9±2.3 years, and females represented 58.9% of the sample. The majority of the students were single (92.8%), living in urban areas (68.5%), and studying in governmental (public) universities (77.0%). Of all students, 51.0% were in their basic years, whereas 49.0% were in their clinical years. Nearly half of the students (53.0%) have relatives in the healthcare field. These were first-degree relatives for 35.9% of the students and second-degree relatives for 25.4% (Table 1).

Of the 13,060 students, 5848 (44.8%) have participated in a research-related activity. Only 31.0% of the students have heard about secondary research. Regarding the types of secondary research known by the students, systematic reviews and meta-analyses were the most frequently identified types (by 20.8% and 18.8% of the students, respectively). About 25% of the students have received training on secondary research, and 16.1% received that training as part of their medical school curriculum. Nearly 13.5% of students have access to subscription databases (Table 2).

Knowledge of secondary research among the students

Of a total secondary research knowledge score of 19, the students' mean score was 2.2±3.2, reflecting an overall very poor level of knowledge. Furthermore, most students (97.3%) had a low knowledge level (Table 2).

Students' level of knowledge and their sociodemographic characteristics

Being a married or widowed student, studying in a private university, studying at the clinical level, and having participated in a research-related activity were significantly associated with higher levels of knowledge (P<0.001) (Table 2).

The students' secondary research knowledge varied significantly by country (P<0.001). Saudi Arabian students had a high score of knowledge (10.8%), followed by students from Egypt (5.4%) and Palestine (5.3%). While only 0.6% of the students from Libya, 2.2% from Sudan, and 2.4% from Yemen achieved a high knowledge score (Supplementary table 1).

Awareness of medical databases

The students were asked about the biomedical databases known to them; PubMed was the most frequently identified one (by 42.9% of the students), followed by Google scholar (36.2%) and Web of Science (15.0%) (Table 3).

Systematic reviews and meta-analyses skills

Regarding the undergraduate medical students' secondary research skills, the most frequent above-average skills were searching online databases (13.3%), followed by the title and abstract screening (10%). However, the least reported above-average skills were statistical meta-analysis (5.9%) and protocol registration (5.7%) (Table 4).

Barriers to conducting systematic reviews

The barriers that the students faced towards conducting systematic reviews were; a lack of knowledge about systematic reviews (47.0%), lack of research exposure and opportunities (28.8%), lack of supervision and guidance (28.5%), lack of time (27.1%), the difficulty of finding person conducting statistical meta-analysis (23.8%), lack of fund (23.2%), limited access to subscriptions database (23.0%), the difficulty of finding experts authors in the systematic review (22.1%), inability to find research question (idea) (20.4%), inability to find full texts for articles (20.3%) and lack of interest (20.1%). (Table 5)

The Practice of secondary research

Out of the total number of students (n=13,060), only 12% (n=1567) have participated in secondary research (Figure 2). The types of research projects students mainly enrolled in were; systematic reviews (62.3%), followed by systematic reviews and meta-analysis (43.3%), then network meta-analysis (33.4%), and lastly, scoping review (28.6%). Of those who have participated in secondary research projects, 471 have published their work. The most common type of publication was systematic review (26.4%), followed by systematic review and meta-analysis (20.1%), network meta-analysis (16.2%), rapid review (13.3%), and scoping review (14.2%). (Table 6)

Among the 934 students who have enrolled in systematic review research projects, the most used database in conducting the systematic review was PubMed (54.9%), followed by Google Scholar (37.9%), Web of Science (28.0%), Scopus (23.3%), Science Direct (21.6%) and Cochrane central for randomized control trials (20.7%). The frequent steps of systematic

reviews that students took part in were; formulating and validating the research question (47.5%), followed by searching online databases (34.3%), data extraction (30.8%), developing and writing the protocol (32.9%) and title and abstract screening (29.9%). RevMan and SPSS version 28 were used by these students for conducting statistical meta-analysis (26.6% and 27.6%, respectively), followed by open meta-analyst (21.1%), R-package in R-software (20.0%), Comprehensive Meta-Analysis Software (CMA) (20.6%), and finally Stata (20.3%). (Table 6)

Discussion

This study tackled the topic of systematic review and meta-analysis popularity among undergraduate medical students in the middle east. The study aimed to identify essential variables affecting the level of knowledge between participants and the perceived barriers to practicing systematic reviews.

With 13060 participating medical students- half of them Sudanese, the mean knowledge score was 2.2 (SD=3.2), reflecting a high percentage of overall low level of knowledge about systematic review (97.3%). This can be owed to the fact that less than one-third of the participants heard of secondary research (31.0%), and less than one-fifth are familiar with systematic reviews (20.8%). Our study showed a low level of knowledge which was found compatible with a study conducted among medical students of six Arab countries, which reported that out of 2989 participants, 91.6% showed poor knowledge towards research as a whole [10].

Of the participants, students at senior years -significantly- had a better knowledge about systematic review than junior students, which is justified as a result of more prolonged exposure to the variable aspects of the medical field. Also, being a married, widowed student, a male, studying in a private university, in the fifth, sixth, or internship year, studying at the clinical level, and participating in a research-related activity were factors related to higher levels of knowledge toward systematic review, which comes in agreement with a study that reported being a student in a top university, having a father or a mother with a college degree and being in the clinical years were significantly associated with higher levels of knowledge towards research [10].

Participating in research was associated with a high level of knowledge about research in a previous study, assessed research analysis among medical residents in Saudi Arabia [12], and knowledge about research undergraduate medical and dental students [13]. This confirms that medical students' involvement in research would enhance their knowledge of research and systematic review. However, across Arab countries, Saudi Arabian students are leading the race with the highest levels of knowledge of systematic review, contrary to Libyan students, who end at the tail. This fact differed from another study that assessed research knowledge in six Arab countries and showed that Jordanian students had a high knowledge score about research and the lowest score for Sudanese students [10]. These findings confirm that knowledge about research and systematic reviews differed across countries.

A previous study reported systemic reviews and meta-analysis were the second most commonly conducted research in Egypt and the third in Jordan and Syria [10]. This could be explained by comparing the awareness and attitude towards medical research and the research fields in general, which gives an insight into the educational system adopted among undergraduate medical students.

The infamous database - "PubMed" – is the most widely used and gives free access to the MEDLINE (Medical Literature Analysis and Retrieval System Online, or MEDLARS Online) database [14]. Among the medical students participating in a study regarding Evidence-based Medicine (EBM) in Sudan, students reported Google to search and Wikipedia as the most common search engines. In contrast, students enrolled in EBM training had a higher level of using PubMed as a search engine [15]. Also, in Hungary, students of medicine and health sciences commonly used Google, followed by Wikipedia, and PubMed, and many students used PubMed [16]. Our participants across countries in this study identified PubMed as the most reputable database, and this explains that familiarity with other databases and the variable types of systematic review is linked with the available opportunities to participate in systematic review projects as well as a stable fund resource, accessibility to databases, and availability of senior guidance, which is considered a problematic issue in the medical education field in the middle-east.

Regarding skills of systematic review, the most advanced skills reported in our study were searching online databases (4.1%), followed by the title and abstract screening (3.3%), and the least below-average skill was in Statistical meta-analysis (80.7%), followed by Protocol registration. Another previous Sudanese study about EBM reported limited skills and experience among medical students in locating professional literature, identifying relevant clinical questions, critical appraisal of scientific papers, and the ability to search online databases [15]. Generally, this explains that systematic review skills varied across participants from all countries.

The lack of knowledge was the most significant barrier facing medical students trying to adopt systematic review in Practice, followed by usual barriers such as lack of time and funds, which is affected by the country's financial status and the institution. Also, a significant barrier was a lack of supervision and guidance and difficulty finding authors with experience in systemic review, raising the flag of a potentially vicious cycle of lack of experience in systematic review and meta-analysis. These findings are supported by a study

conducted in Saudi Arabia among senior medical students, who reported that lack of professional supervision, training courses, and time and funds were the main obstacles to conducting research [17]. Another study among medical science students in Iran identified lack of funding, lack of guidance, limitation in time, and lack of knowledge and skill of research as the most significant barriers toward research [18]. Our study and previous studies showed clearly similar barriers that prevent participants from engaging in research and systematic review, which need a quick solution for this problem to enhance and improve the knowledge and Practice of systematic review.

Regarding implementing systematic review in Practice, only about 12% of the participating medical students took a role in such a project, with the systematic review being more popular than meta-analysis and scope review. This low level of Practice is consistent with the low level of knowledge about systematic review. A previous study in Saudi Arabia reported that the most popular research projects were case reports, retrospective, and prospective clinical studies, and cross-sectional and review articles [17]. Another study reported the most common type of studies to be cross-sectional, followed by case reports and then systematic reviews and meta-analyses [10]. These findings from previous studies confirm the low Practice of systematic review in our study.

This is the first cross-sectional study to tackle an issue concerning systematic review among medical students. The study is generalizable as it covered the majority of middle east countries and included a large sample size. The findings of this study will help -tremendously- in solving the issue of knowledge ad practice of systematic review in undergraduate medical students.

However, this study didn't assess the quality of learning secondary research, which may affect the percentage of knowledge and Practice in each country. Also, non-probability sampling jeopardizes the randomization of the sampling and puts a risk of selection bias.

Conclusion

Knowledge and Practice of systematic review processes among medical students were found to be low. Several barriers hindered undergraduate medical students from conducting systematic reviews. The necessity to adopt secondary research materials in curriculums is highlighted by the finds of this study, as systematic reviews are becoming a postgraduate prerequisite worldwide.

The findings of this study highlight the need to integrate secondary research materials among the curricula of medical schools and provide training and capacity building to strengthen the medical workforce's capability to practice in an evidence-based manner. Projects of peer-teaching are also promising, as it provides a chance for learning by Practice. Professionals in systematic reviewing and experienced data analysts should be provided in the teaching staff to overcome the supervision issue.

Resources denote an enormous concern for undergraduate students. Libraries that require a subscription need to be funded by educational institutions to provide unlimited access for students.

Declaration

Sudan Analytics Research Group team of collaborators: Abdelrahim A Ahmed, Abdelrhman mohamed abdelrady, Abdelrhman Muwafaq Janem, Abdelrhman Waleed Elshenawy, Abdulghani Arja, Abdullah alshogaa, Abdullah Nasser Ali Hamilah, Abdurahim Mustafa Alaswad, Abeer S. El-Shawady, Abubakr Muhammad, Afaf Ahmad, Afnan Mousa Alhawsawi, Ahmad Ali Essa, Ahmed Khalid Yousif Mustafa, Al Zahraa Ali, Alaa Amr, Albushra A. Adam, Ali Alkhamis, Amna Altayeb Alhaj Ahmed, Aml Mostafa Farid, Amr Mahmoud Abdelraoof, Asma Ali alarabi, Asmaa H Alzabadiah, Awab Osman Ahmed Osman, Aya Ahmed Mohamed, Aya.M.abbas, Bashar Bazkke, DarElslam ELawad Osman, Deena faraj abdulali altarjami, Douaa Effat Belal, Ebtihal Mohammed Albasheir, Elsayed Hammad, Enaam alabed ahmed adam, Entisar Abdalla Zin Elabdein Ahmed, Esra Magzoub Ibrahem Ahmed, Esraa Salaheldin Ahmed Alfadul, Essraa ali alhudhairy, Faris Hamed, Ghadeer Ahmed Alhawsawi, Hagar K Elgazar, Haifa saif aldeen Alsaid humid, Haneen A. Banihani, Hassan Elamin Hassan Elamin, Hawa Essa Mohammed, Haya Deeb, Heba Alwaa, Hosam Khalouf, Hussein Mohammed Hussein Ghalib, ihcene Telaidji, Intisar Bubaker Owhida, Ismail Adam, Khadija Tariq Habib, Khaled Albakri, Khaled Tawfeeq, Khalid Elsir Idris, Khawla Alshahrani, Khetam Mohamed Saleh Adah, Khulod Ali Bokir, Kinda ahmed hardan, Lubna Hossam Aloufi, Lulyah almallah, Maazouz Bensalem, Maisa Nazzal Ayman Nazzal, Majid H Ali, Makhlouf Sofia, Manar Kahoul, Mawahib Elsheikh, Mawda Jumma, Mazin Mohammed Osman, Moath Almekhlafi, Moath Rushdi Atiani, Mobtahel Modather mohammed omer, Moftah Imhamed Moftah Himeda, Mohamed Abdelmonem, Mohamed Baklola, Mohamed Baraka, Mohamed Faroug Ali Yassin, Mohamed Gamal Eldow Abbas, Mohamed Ibrahem Baklola, Mohammad Abdulrahman Aljundi, Mohammad Saleh Alwajih, Mohammed Amir Rais, Mohmmad awd alsyed ahmad abbass, Monzer Elgamal, Malaz Omer Altegani Mohammed, Muhammad Sayed Muhammad Masoud, Mutwakel Ali Mohammed, Nagwa yassin Mohammad Taha, Nisreen Abufaris, Noon Isam Eldin Hasssan Hagmusa, Noura Ghazi Abu Lehia, Nourhan Elsamahy, Omar Abdulraheem Baqais, Omar Riffi, Omar S. Aldhafeeri, Omer A. Idrees, Qosay Sami Jabr, Raghad Nabeel Sadi Saleh, Rahaf mutlaq alotaibi, Rasha Nasser Hasnah, Rawan AbdAlwahd Mohammed Osman, Reema Saleh Zayed Alamri, Riham Mohammed Osman Mohammed, Roaa B. Albashir, Roua Arian, Ruwayda Abdulmutalib mawloud, Selema Nabeel Belgasem, Shahd T. Idais, Somayya Tabsho, Thekra Fawaz Albeshri, Turfa Moudarres, Waad Abu Elbeada, Wafaa Eshag Moheyeldeen, Yousef Abdullah Aldreweesh, Ziad Alahmad, Rawa Naimat, Majedah A. Alakayleh, Shahed N.

Abughanam, Mallak I. Alja'fari, Razan M. Altarawaneh, Ihab A. Alsalamin, Duoaa Albelal, Ahmad Aissa.

Consent for publication: No personal data was collected from the participants.

Disclosure statement: No potential conflict of interest was reported by the author(s).

Availability of data and materials: The datasets generated and/or analyzed during the current study are not publicly available due to privacy but are available from the corresponding author on reasonable request.

Competing interest: This manuscript has been published as E-poster and in AMEE 2022 abstracts book at Association for Medical Education in Europe (AMEE) Conference: Redefining Health Professions Education Together in August 2022. The authors declare that they have no financial or non-financial competing interests.

Funding: None.

Authors' contribution: EAH contributed to the idea of the research project, conception, and design of the study, project administration, supervision, questionnaire design, analysis plan, data analysis, creation, and interpretation, writing the manuscript, and drafting the article. **WE** contributed to the study's data collection, conception, and design, project administration, supervision, questionnaire design, writing the manuscript, and drafting the article. **ASA, AAA:** contributed to the data collection, recruiting collaborators, questionnaire design, writing the manuscript, and revising the article. **KAHMA, GEMA, MSS, MFE:** contributed to the data collection, writing the manuscript, and revising the article. **A.A.:** contributed to the data collection and revision of the article**. HAS, H.A., AYB, AWMJ, TM, OA, MIA, MZBA, and AA:** contributed to the data collection and revision of the article. All authors revised the manuscript and approved it for publication.

Acknowledgments: The authors would like to thank the research agency: "Sudan Analytics for Research and Statistics" (Links: https://www.facebook.com/Sudan.Analytics/; https://www.linkedin.com/company/76092484/; https://twitter.com/Sudananalytics; https://telegram.me/Sudananalytics) for their help in our research. Also, we would like to thank undergraduate medical students for participating in this study.

Additional files:

1- Supplementary table 1 (docx.) includes the level of knowledge and Practice of systematic review according to countries.

References

1. Mengist W, Soromessa T, Legese G. Method for conducting systematic literature review and meta-analysis for environmental science research. *MethodsX*. 2020;7:100777. doi:10.1016/j.mex.2019.100777
2. Krnic Martinic M, Pieper D, Glatt A, Puljak L. Definition of a systematic review used in overviews of systematic reviews, meta-epidemiological studies and textbooks. *BMC Medical Research Methodology*. 2019;19(1):203. doi:10.1186/s12874-019-0855-0
3. Oliver K, Innvar S, Lorenc T, Woodman J, Thomas J. A systematic review of barriers to and facilitators of the use of evidence by policymakers. *BMC Health Services Research*. 2014;14(1):2. doi:10.1186/1472-6963-14-2
4. Gopalakrishnan S, Ganeshkumar P. Systematic Reviews and Meta-analysis: Understanding the Best Evidence in Primary Healthcare. *Journal of family medicine and primary care*. 2013;2(1):9-14. doi:10.4103/2249-4863.109934
5. Munn Z, Peters MDJ, Stern C, Tufanaru C, McArthur A, Aromataris E. Systematic review or scoping review? Guidance for authors when choosing between a systematic or scoping review approach. *BMC Medical Research Methodology*. 2018;18(1):143. doi:10.1186/s12874-018-0611-x
6. Szajewska H. Evidence-Based Medicine and Clinical Research: Both Are Needed, Neither Is Perfect. *Annals of nutrition & metabolism*. 2018;72 Suppl 3:13-23. doi:10.1159/000487375
7. Herrera Ortiz AF, Cadavid Camacho E, Cubillos Rojas J, et al. A Practical Guide to Perform a Systematic Literature Review and Meta-analysis. *Principles and Practice of Clinical Research Journal*. 2021;7(4):47-57. doi:10.21801/ppcrj.2021.74.6
8. Boaz A, Baeza J, Fraser A, European Implementation Score Collaborative Group (EIS). Effective implementation of research into practice: an overview of systematic reviews of the health literature. *BMC research notes*. 2011;4:212. doi:10.1186/1756-0500-4-212
9. Wallace J, Nwosu B, Clarke M. Barriers to the uptake of evidence from systematic reviews and meta-analyses: a systematic review of decision makers' perceptions. *BMJ Open*. 2012;2(5):e001220. doi:10.1136/bmjopen-2012-001220
10. Assar A, Matar SG, Hasabo EA, et al. Knowledge, attitudes, practices and perceived barriers towards research in undergraduate medical students of six Arab countries. *BMC Medical Education*. 2022;22(1):44. doi:10.1186/s12909-022-03121-3

11. Tricco AC, Cardoso R, Thomas SM, et al. Barriers and facilitators to uptake of systematic reviews by policy makers and health care managers: a scoping review. *Implementation Science*. 2015;11(1):4. doi:10.1186/s13012-016-0370-1
12. Albumijdad J, Alismail A, Albesher M, Alhajri A. Attitude, knowledge, and barriers towards research analysis and writing in Saudi Arabia. *Journal of Family Medicine and Primary Care*. 2022;11(5):2123. doi:10.4103/jfmpc.jfmpc_1991_21
13. Kyaw Soe H, Than N, Lwin H, Nu Htay M, Phyu K, Abas A. Knowledge, attitudes, and barriers toward research: The perspectives of undergraduate medical and dental students. *Journal of Education and Health Promotion*. 2018;7(1):23. doi:10.4103/jehp.jehp_61_17
14. About - PubMed. Accessed October 29, 2022. https://pubmed.ncbi.nlm.nih.gov/about/
15. Hasabo EA, Elnaiem W, Ali AY, et al. Evidence-based medicine (EBM) for undergraduate medical students in Sudan: sources of information, knowledge about terms, skills related to EBM and attitude toward EBM in Sudan. *BMC Medical Education*. 2021;21(1):470. doi:10.1186/s12909-021-02902-6
16. Csertő M, Berényi K, Decsi T, Lohner S. Self-reported attitudes, knowledge and skills of using evidence-based medicine in daily health care practice: A national survey among students of medicine and health sciences in Hungary. Bianchi C, ed. *PLOS ONE*. 2019;14(12):e0225641. doi:10.1371/journal.pone.0225641
17. AlGhamdi KM, Moussa NA, AlEssa DS, AlOthimeen N, Al-Saud AS. Perceptions, attitudes and practices toward research among senior medical students. *Saudi Pharmaceutical Journal*. 2014;22(2):113-117. doi:10.1016/j.jsps.2013.02.006
18. Memarpour M, Fard AP, Ghasemi R. Evaluation of attitude to, knowledge of and barriers toward research among medical science students. *Asia Pacific Family Medicine*. 2015;14(1):1. doi:10.1186/s12930-015-0019-2

Tables and figures legends:

Figure 1: Number of participants in included Arabs countries.

Figure 2: Enrolment or practicing a secondary review among participants.

Supplementary table 1: Level of knowledge and Practice of systematic review according to countries.

Table 1: Baseline characteristics and difference in the level of knowledge for systematic review and Practice of secondary research in undergraduate medical students.

Table 2: Level of knowledge about systematic reviews in undergraduate medical students.

Table 3: Medical databases being known by undergraduate medical students.

Table 4: Rating skill of meta-analysis in undergraduate medical students

Table 5: Barrier to conducting systematic reviews in undergraduate medical students.

Table 6: Practice of systematic reviews in undergraduate medical students.

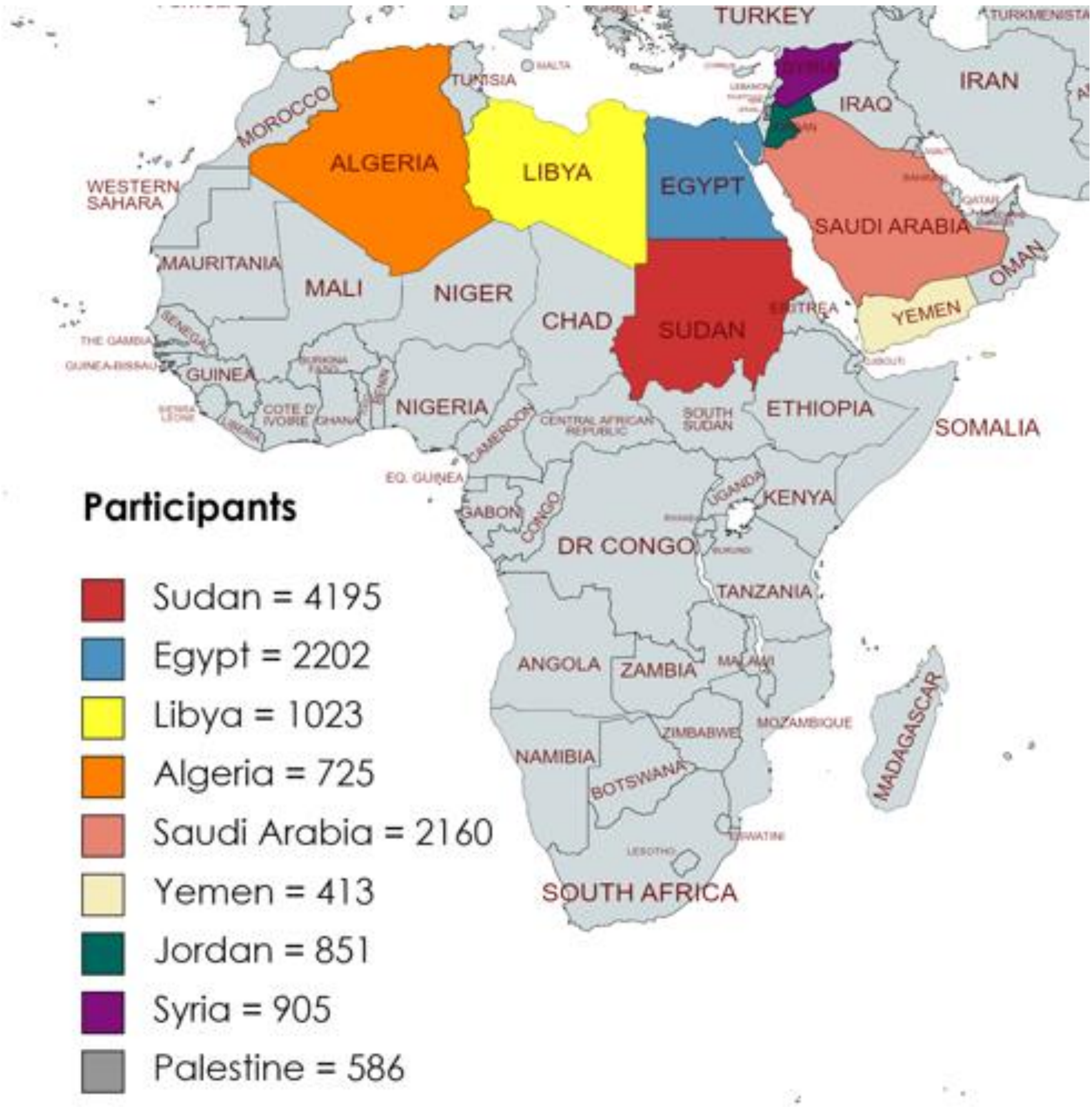

Figure 1: Number of participants in included Arabs countries.

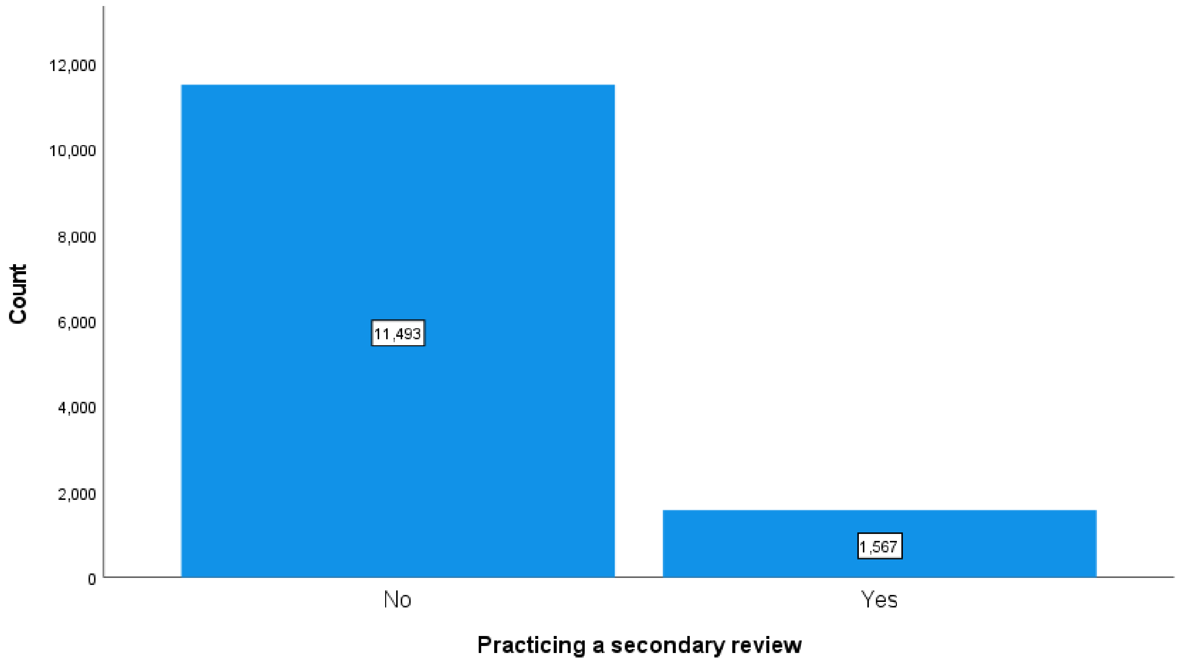

Figure 2: Enrolment or practicing a secondary review among participants.

Table 1: Baseline characteristics and difference in the level of knowledge for systematic review and Practice of secondary research in undergraduate medical students.

**Variables**	**N**	**Overall**, N = 13,060[1]	**Level of knowledge**		**p-value**[2]	**Enrolled or practiced in secondary research**		**p-value**[2]
			**High**, N = 563[1]	**Low**, N = 12,497[1]		**No**, N = 11,493[1]	**Yes**, N = 1,567[1]	
**Age, years**	13,002	21.9 ± 2.3	22.6 ± 2.4	21.9 ± 2.3	**<0.001**	21.9 ± 2.3	22.3 ± 2.4	**<0.001**
**Gender**	13,060				**0.038**			**<0.001**
Female		7,694 (58.9%)	308 (54.7%)	7,386 (59.1%)		6,867 (59.7%)	827 (52.8%)	
Male		5,366 (41.1%)	255 (45.3%)	5,111 (40.9%)		4,626 (40.3%)	740 (47.2%)	
**Residence**	13,060				0.2			**<0.001**
Rural		4,109 (31.5%)	190 (33.7%)	3,919 (31.4%)		3,521 (30.6%)	588 (37.5%)	
Urban		8,951 (68.5%)	373 (66.3%)	8,578 (68.6%)		7,972 (69.4%)	979 (62.5%)	
**Marital status**	13,060				**<0.001**			**<0.001**
Divorced		93 (0.7%)	10 (1.8%)	83 (0.7%)		67 (0.6%)	26 (1.7%)	
Married		756 (5.8%)	66 (11.7%)	690 (5.5%)		647 (5.6%)	109 (7.0%)	
Single		12,125 (92.8%)	466 (82.8%)	11,659 (93.3%)		10,704 (93.1%)	1,421 (90.7%)	
Widowed		86 (0.7%)	21 (3.7%)	65 (0.5%)		75 (0.7%)	11 (0.7%)	
**Type of your university**	13,060				**0.037**			**0.001**

Private		3,006 (23.0%)	150 (26.6%)	2,856 (22.9%)		2,595 (22.6%)	411 (26.2%)	
Public (Governmental)		10,054 (77.0%)	413 (73.4%)	9,641 (77.1%)		8,898 (77.4%)	1,156 (73.8%)	
**Year of Study**	13,060				**<0.001**			**<0.001**
First-year		1,532 (11.7%)	38 (6.7%)	1,494 (12.0%)		1,397 (12.2%)	135 (8.6%)	
Second-year		2,412 (18.5%)	70 (12.4%)	2,342 (18.7%)		2,204 (19.2%)	208 (13.3%)	
Third-year		2,666 (20.4%)	110 (19.5%)	2,556 (20.5%)		2,377 (20.7%)	289 (18.4%)	
Fourth-year		2,451 (18.8%)	107 (19.0%)	2,344 (18.8%)		2,141 (18.6%)	310 (19.8%)	
Fifth-year		2,015 (15.4%)	109 (19.4%)	1,906 (15.3%)		1,716 (14.9%)	299 (19.1%)	
Sixth-year		1,130 (8.7%)	74 (13.1%)	1,056 (8.5%)		951 (8.3%)	179 (11.4%)	
Internship		854 (6.5%)	55 (9.8%)	799 (6.4%)		707 (6.2%)	147 (9.4%)	
**Current level in medical faculty**	13,060				**<0.001**			**<0.001**
Basic		6,666 (51.0%)	233 (41.4%)	6,433 (51.5%)		6,033 (52.5%)	633 (40.4%)	
Clinical		6,394 (49.0%)	330 (58.6%)	6,064 (48.5%)		5,460 (47.5%)	934 (59.6%)	
**Do you have a family member (parent, brothers, sisters, etc.) working in healthcare?**	13,060	6,925 (53.0%)	347 (61.6%)	6,578 (52.6%)	**<0.001**	5,927 (51.6%)	998 (63.7%)	**<0.001**

**First-degree (Father, mother, brother, sister)**	13,060	4,688 (35.9%)	257 (45.6%)	4,431 (35.5%)	**<0.001**	3,934 (34.2%)	754 (48.1%)	**<0.001**
**Second-degree (Grandfather, grandmother, uncle, aunt, cousin,..etc.)**	13,060	3,322 (25.4%)	171 (30.4%)	3,151 (25.2%)	**0.006**	2,919 (25.4%)	403 (25.7%)	0.8
**Have you ever participated in a research-related activity (class/ workshop/project, etc.)?**	13,060	5,848 (44.8%)	440 (78.2%)	5,408 (43.3%)	**<0.001**	4,703 (40.9%)	1,145 (73.1%)	**<0.001**

[1] n (%); Mean ± SD

[2] Pearson's Chi-squared test; Two Sample t-test; Fisher's exact test

Table 2: Level of knowledge about systematic reviews in undergraduate medical students. (n=13,060

Variables	N	Overall, N = 13,060[1]	Level of knowledge: High, N = 563[1]	Level of knowledge: Low, N = 12,497[1]	p-value[2]
**Have you heard about secondary research?**	13,060	4,052 (31.0%)	467 (82.9%)	3,585 (28.7%)	**<0.001**
**If yes, what types of secondary research do you know?**					
Systematic review	13,060	2,713 (20.8%)	317 (56.3%)	2,396 (19.2%)	**<0.001**
Systematic review and meta-analysis	13,060	2,456 (18.8%)	310 (55.1%)	2,146 (17.2%)	**<0.001**
Network meta-analysis	13,060	992 (7.6%)	207 (36.8%)	785 (6.3%)	**<0.001**
Scoping review	13,060	561 (4.3%)	143 (25.4%)	418 (3.3%)	**<0.001**
Narrative review	13,060	814 (6.2%)	169 (30.0%)	645 (5.2%)	**<0.001**
**Have you attended or received training on systematic reviews?**	13,060	3,275 (25.1%)	425 (75.5%)	2,850 (22.8%)	**<0.001**
**If yes (attended or received training on systematic reviews), what were your training sources?**					
Medical school (in the curriculum)	13,060	2,105 (16.1%)	262 (46.5%)	1,843 (14.7%)	**<0.001**
Physical extracurricular training	13,060	791 (6.1%)	157 (27.9%)	634 (5.1%)	**<0.001**
Online extracurricular training	13,060	1,112 (8.5%)	202 (35.9%)	910 (7.3%)	**<0.001**
Peer teaching	13,060	521 (4.0%)	111 (19.7%)	410 (3.3%)	**<0.001**
**Do you have access to subscription databases (Paid databases) used in systematic review? (Yes)**	13,060	1,758 (13.5%)	240 (42.6%)	1,518 (12.1%)	**<0.001**
**Questions about knowledge**					
**The highest level of evidence in research is? (Systematic review and meta-analysis)**	13,060	3,536 (27.1%)	340 (60.4%)	3,196 (25.6%)	**<0.001**
**On which of the following can you register a systematic review protocol? (Prospero)**	13,060	829 (6.3%)	341 (60.6%)	488 (3.9%)	**<0.001**

**The reporting guideline for systematic reviews is? (Prisma)**	13,060	985 (7.5%)	348 (61.8%)	637 (5.1%)	**<0.001**
**Which of the following programs can be used in conducting a meta-analysis? (You can choose more than one answer)**					
**RevMan**	13,060				**<0.001**
I don't know		10,693 (81.9%)	54 (9.6%)	10,639 (85.1%)	
No		672 (5.1%)	34 (6.0%)	638 (5.1%)	
Yes (Correct answer)		1,695 (13.0%)	475 (84.4%)	1,220 (9.8%)	
**R software**	13,060				**<0.001**
I don't know		10,262 (78.6%)	50 (8.9%)	10,212 (81.7%)	
No		1,073 (8.2%)	99 (17.6%)	974 (7.8%)	
Yes (Correct answer)		1,725 (13.2%)	414 (73.5%)	1,311 (10.5%)	
**Stata**	13,060				**<0.001**
I don't know		10,523 (80.6%)	76 (13.5%)	10,447 (83.6%)	
No		956 (7.3%)	83 (14.7%)	873 (7.0%)	
Yes (Correct answer)		1,581 (12.1%)	404 (71.8%)	1,177 (9.4%)	
**SPSS version 23**	13,060				**<0.001**
I don't know		9,411 (72.1%)	70 (12.4%)	9,341 (74.7%)	
No (Correct answer)		807 (6.2%)	146 (25.9%)	661 (5.3%)	
Yes		2,842 (21.8%)	347 (61.6%)	2,495 (20.0%)	
**Which of the following are quality assessment tools used in systematic reviews? (You can choose more than one answer)**	0	0 (NA%)	0 (NA%)	0 (NA%)	
**Newcastle-Ottawa Scale**	13,060				**<0.001**

I don't know		10,801 (82.7%)	59 (10.5%)	10,742 (86.0%)	
No		674 (5.2%)	64 (11.4%)	610 (4.9%)	
Yes (Correct answer)		1,585 (12.1%)	440 (78.2%)	1,145 (9.2%)	
**PRISMA**	13,060				**<0.001**
I don't know		10,563 (80.9%)	48 (8.5%)	10,515 (84.1%)	
No (Correct answer)		1,160 (8.9%)	250 (44.4%)	910 (7.3%)	
Yes		1,337 (10.2%)	265 (47.1%)	1,072 (8.6%)	
**Cochrane risk of bias tool**	13,060				**<0.001**
I don't know		10,306 (78.9%)	25 (4.4%)	10,281 (82.3%)	
No		918 (7.0%)	86 (15.3%)	832 (6.7%)	
Yes (Correct answer)		1,836 (14.1%)	452 (80.3%)	1,384 (11.1%)	
**NIH tool**	13,060				**<0.001**
I don't know		10,775 (82.5%)	91 (16.2%)	10,684 (85.5%)	
No		1,013 (7.8%)	116 (20.6%)	897 (7.2%)	
Yes (Correct answer)		1,272 (9.7%)	356 (63.2%)	916 (7.3%)	
**Meta-analysis**	13,060				**<0.001**
I don't know		9,658 (74.0%)	79 (14.0%)	9,579 (76.7%)	
No (Correct answer)		818 (6.3%)	191 (33.9%)	627 (5.0%)	
Yes		2,584 (19.8%)	293 (52.0%)	2,291 (18.3%)	
**What is the name of this figure? (Forest plot)**	13,060	1,247 (9.5%)	329 (58.4%)	918 (7.3%)	**<0.001**

**Which of the following tests can be used to assess for heterogeneity across the studies in meta-analysis? (You can choose more than one answer)**	0	0 (NA%)	0 (NA%)	0 (NA%)	
**Chi-squared test**	13,060				**<0.001**
I don't know		10,003 (76.6%)	29 (5.2%)	9,974 (79.8%)	
No		660 (5.1%)	58 (10.3%)	602 (4.8%)	
Yes (Correct answer)		2,397 (18.4%)	476 (84.5%)	1,921 (15.4%)	
**T-test**	13,060				**<0.001**
I don't know		10,045 (76.9%)	77 (13.7%)	9,968 (79.8%)	
No (Correct answer)		1,215 (9.3%)	261 (46.4%)	954 (7.6%)	
Yes		1,800 (13.8%)	225 (40.0%)	1,575 (12.6%)	
**I-squared test**	13,060				**<0.001**
I don't know		10,512 (80.5%)	60 (10.7%)	10,452 (83.6%)	
No		950 (7.3%)	88 (15.6%)	862 (6.9%)	
Yes (Correct answer)		1,598 (12.2%)	415 (73.7%)	1,183 (9.5%)	
**Inverse variance**	13,060				**<0.001**
I don't know		10,880 (83.3%)	135 (24.0%)	10,745 (86.0%)	
No (Correct answer)		979 (7.5%)	223 (39.6%)	756 (6.0%)	
Yes		1,201 (9.2%)	205 (36.4%)	996 (8.0%)	
**Which of the following models is used in the presence of significant heterogeneity? (Random-effect model)**	13,060	2,107 (16.1%)	341 (60.6%)	1,766 (14.1%)	**<0.001**

**To compare multiple treatments using direct comparisons of interventions within randomized controlled trials and indirect comparisons across trials based on a common comparator we use? (Network meta-analysis)**	13,060	954 (7.3%)	284 (50.4%)	670 (5.4%)	**<0.001**
**The score of knowledge (Total =19)**	13,060	2.2 ± 3.2	11.5 ± 1.8	1.7 ± 2.5	**<0.001**

[1] n (%); Mean ± SD

[2] Pearson's Chi-squared test; Two Sample t-test; Fisher's exact test

Table 3: Medical databases being known by undergraduate medical students.

Variables	N	Overall, N = 13,060[1]	Level of knowledge		p-value[2]
			High, N = 563[1]	Low, N = 12,497[1]	
**Which of the following databases do you know? (if you don't know, leave it empty) (You can choose more than one answer)**	13,060				
PubMed		5,601 (42.9%)	408 (72.5%)	5,193 (41.6%)	**<0.001**
Ovid		995 (7.6%)	215 (38.2%)	780 (6.2%)	**<0.001**
PsycINFO		842 (6.4%)	162 (28.8%)	680 (5.4%)	**<0.001**
Web of Science		1,958 (15.0%)	276 (49.0%)	1,682 (13.5%)	**<0.001**
Scopus		1,733 (13.3%)	256 (45.5%)	1,477 (11.8%)	**<0.001**
Cochrane central for randomized control trials		1,374 (10.5%)	218 (38.7%)	1,156 (9.3%)	**<0.001**
Science direct.		1,706 (13.1%)	218 (38.7%)	1,488 (11.9%)	**<0.001**
Google Scholar		4,732 (36.2%)	293 (52.0%)	4,439 (35.5%)	**<0.001**
CINAHL (Cumulative Index to Nursing and Allied Health Literature)		635 (4.9%)	117 (20.8%)	518 (4.1%)	**<0.001**
Health & Medical Complete (ProQuest)		1,010 (7.7%)	130 (23.1%)	880 (7.0%)	**<0.001**
Health Collection (informit)		759 (5.8%)	128 (22.7%)	631 (5.0%)	**<0.001**
Clinicaltrial.gov		1,012 (7.7%)	179 (31.8%)	833 (6.7%)	**<0.001**
African Journals OnLine (AJOL)		787 (6.0%)	91 (16.2%)	696 (5.6%)	**<0.001**
Embase		17 (0.1%)	11 (2.0%)	6 (0.0%)	**<0.001**
Clinical Key		2 (0.0%)	0 (0.0%)	2 (0.0%)	>0.9

[1] n (%)

[2] Pearson's Chi-squared test; Fisher's exact test

Table 4: Rating skill of meta-analysis in undergraduate medical students

	Poor	Limited experience	Average	Above average	Advanced
**How would you rate your skills in the following steps of meta-analysis? (You can choose either: Poor, Limited experience, Average, Above average, or Advanced)**					
Formulate a question and its validation	6,851 (52.5%)	2,630 (20.1%)	2,462 (18.9%)	745 (5.7%)	372 (2.8%)
Develop and write protocol (eligibility criteria, keyword search strategy)	7,019 (53.7%)	2,998 (23.0%)	2,140 (16.4%)	631 (4.8%)	272 (2.1%)
Creating search string	7,432 (56.9%)	2,594 (19.9%)	2,188 (16.8%)	584 (4.5%)	262 (2.0%)
Searching online databases	6,063 (46.4%)	2,549 (19.5%)	2,712 (20.8%)	1,195 (9.2%)	541 (4.1%)
Protocol registration	7,863 (60.2%)	2,494 (19.1%)	1,981 (15.2%)	469 (3.6%)	253 (1.9%)
Title and abstract screening	6,839 (52.4%)	2,587 (19.8%)	2,332 (17.9%)	871 (6.7%)	431 (3.3%)
Full-text screening	7,053 (54.0%)	2,560 (19.6%)	2,309 (17.7%)	757 (5.8%)	381 (2.9%)
Data extraction	6,864 (52.6%)	2,656 (20.3%)	2,354 (18.0%)	811 (6.2%)	375 (2.9%)
Quality assessment of included studies	7,431 (56.9%)	2,627 (20.1%)	2,096 (16.0%)	619 (4.7%)	287 (2.2%)
Statistical meta-analysis	8,042 (61.6%)	2,498 (19.1%)	1,754 (13.4%)	524 (4.0%)	242 (1.9%)
Manuscript writing	7,659 (58.6%)	2,390 (18.3%)	2,091 (16.0%)	593 (4.5%)	327 (2.5%)

Table 5: Barrier to conducting systematic reviews in undergraduate medical students.

Variables	**Overall**, N = 13,060[1]
**Which of the following constitutes a barrier for you towards conducting systematic reviews? (You can choose more than one answer, and if there are no barriers, leave this question empty)**	
Lack of knowledge about systematic review	6,136 (47.0%)
Inability to find full text for articles	2,656 (20.3%)
Inability to find a research question (Idea)	2,664 (20.4%)
Lack of research exposure and opportunities	3,756 (28.8%)
Limited access to subscription databases	3,003 (23.0%)
Lack of supervision and guidance	3,719 (28.5%)
Lack of interest	2,619 (20.1%)
Lack of time	3,544 (27.1%)
Lack of fund	3,036 (23.2%)
The difficulty of finding experts authors in the systematic review	2,890 (22.1%)
The difficulty of finding a person to conduct a statistical meta-analysis	3,111 (23.8%)

[1] n (%)

Table 6: Practice of systematic reviews in undergraduate medical students.

**Variables**	**N**	**Overall**, N = 13,060[1]
**Have you ever enrolled in or participated in a systematic review? (Yes)**	13,060	1,567 (12.0%)
**If yes, which type of systematic reviews have you worked on? (You can choose more than one answer)**	1,567	
Systematic review		977 (62.3%)
Systematic review and meta-analysis		679 (43.3%)
Network meta-analysis		524 (33.4%)
Scoping review		448 (28.6%)
Rapid review		1 (0.1%)
**Have you ever published secondary research? (Yes)**	1,567	471 (30.1%)
**If you have systematic review publications, please specify the type for systematic review. (if you don't have it, leave it empty)**	1,567	
Systematic review		414 (26.4%)
Systematic review meta-analysis		315 (20.1%)
Network meta-analysis		254 (16.2%)
Scoping review		223 (14.2%)
Rapid review		208 (13.3%)
Others		220 (14.0%)
**Which databases are you using in the systematic review? (You can choose more than one answer)**	1,567	0 (NA%)
PubMed		860 (54.9%)
Ovid		286 (18.3%)
PsycINFO		256 (16.3%)
Web of Science		439 (28.0%)
Scopus		365 (23.3%)
Cochrane central for randomized control trials		325 (20.7%)
Science direct		338 (21.6%)
Google Scholar		594 (37.9%)
CINAHL (Cumulative Index to Nursing and Allied Health Literature)		213 (13.6%)
Health & Medical Complete (ProQuest)		267 (17.0%)
Health Collection (informit)		250 (16.0%)
Clinicaltrial.gov		284 (18.1%)
African Journals Online (AJOL)		258 (16.5%)
**At which step of systematic review have you participated? (You can choose more than one answer)**	1,567	0 (NA%)
Formulate a question and its validation		744 (47.5%)
Develop and write protocol (eligibility criteria, keyword search strategy)		515 (32.9%)
Creating search string		429 (27.4%)

Searching online databases		538 (34.3%)
Protocol registration		324 (20.7%)
Title and abstract screening		469 (29.9%)
Full-text screening		449 (28.7%)
Data extraction		482 (30.8%)
Quality assessment of included studies		364 (23.2%)
Statistical meta-analysis		343 (21.9%)
Manuscript writing		389 (24.8%)
**If you have done a statistical meta-analysis, which programs have you used? (You can choose more than one answer)**	1,567	0 (NA%)
RevMan		417 (26.6%)
Comprehensive Meta-Analysis Software (CMA)		323 (20.6%)
R packages in R software		314 (20.0%)
SPSS version 28		433 (27.6%)
Stata		318 (20.3%)
Open meta-analyst		330 (21.1%)

[1] n (%)
