# Supplementary material for: Knowledge and preventive barriers towards conducting systematic review among undergraduate medical students of Arab countries: A multi country online survey
Source: PLoS One. 2025 Aug 18;20(8):e0329827. doi: 10.1371/journal.pone.0329827 (PMC12360549; doi:10.1371/journal.pone.0329827)
Supplement: Attachment — Submitted filename: Review comments.docx [file pone.0329827.s003.docx]

**Review comments**

**General comments**

Authors are highly commended for conceptualizing this study and going ahead to conduct and also present their findings for publication. I must say that the study is of immense relevance and valuable.

While I commend the Authors for their great efforts, I am also of the opinion that some aspects of the manuscript should be revised before the manuscript could be accepted for publication.

Authors should take note of the following observations.

1. The manuscript should be reviewed critically for minor errors of grammar. Authors should delete the comment on PUBMED as an infamous database since it is a mistake. In line 142 under Introduction, Authors used ‘our study’.
2. Description of the outcome variable. The main outcome variable for the study included knowledge and practice of systematic review. While knowledge of systematic review was defined, practice was not. In as much, I will advise the Authors to use ≥50% of correct responses to the 19 variables used to determine knowledge as cut off for determining good knowledge of systematic review. In some instances Authors introduced secondary research in the manuscript and this appeared to run parallel to the main outcome variables. This should be clarified.
3. The outcome variables should be categorized into good and poor knowledge of systematic review and good and poor practice of systematic review.
4. With such a large sample size as included in this study, Authors should endeavor to include binary logistic regression analysis in this study. This should be applied for determining the predictors of good knowledge and good practice of systematic review among the respondents. This aspect of the result should be included in the abstract.
5. The presentation of the results should be reviewed to enhance the understanding of readers. For example, there is no presentation of the proportion of respondents that had good knowledge and good practice of systematic review.
6. Authors should be mindful of use of capital letters.
7. Authors should make statements inside the tables and avoid the use of question marks.

**Other comments**

Authors should consider this minor revision of the study title:

**Knowledge, practice and preventive barriers towards conducting systematic reviews among undergraduate medical students of Arab countries: A multi country online survey**

**Abstract**

**Background**

Replace of with among..

**Methods**

Knowledge about systematic reviews was assessed using 19 variables

Instead of using numbers, use proportions to determine good knowledge like ≥50% of correct responses.

Nothing was said about practice.

**Results**

Use majority instead of most

Why were factors affecting knowledge and practice not included?

**Participants**

Why was the age limitation necessary?

Revise the exclusion criteria as one has to be included before the individual could be excluded.

**Use study instrument** instead of instruments used to measure the variables of interest in the study.

Be specific on number of variables used to assess practice of systematic review and how the outcome variable was determined.

**Statistical analysis**

Use frequencies and proportions for categorical variables

**Results**

**Participants’ characteristics**

The proportion, 53% cannot be regarded as nearly half. This should be reviewed.

**Discussion**

Revise the phrase, infamous database……

The conclusion should be in one paragraph.
