# Supplementary material for: Knowledge and preventive barriers towards conducting systematic review among undergraduate medical students of Arab countries: A multi country online survey
Source: PLoS One. 2025 Aug 18;20(8):e0329827. doi: 10.1371/journal.pone.0329827 (PMC12360549; doi:10.1371/journal.pone.0329827)
Supplement: Attachment — Submitted filename: Point-to-point response R1 PLOS (sudan analytics) - Copy.docx [file pone.0329827.s005.docx]

**Point-by-point responses**

**Title of Article:** Knowledge, practice, and perceived barriers towards conducting systematic reviews in undergraduate medical students of Arab countries: A multi-country online survey

**Name of the Corresponding Author:** Elfatih A. Hasabo

**Email Address of the Corresponding Author:** elfatih.ahmed.hasabo@gmail.com

**Journal Requirements:**

When submitting your revision, we need you to address these additional requirements.

1. Please ensure that your manuscript meets PLOS ONE's style requirements, including those for file naming. The PLOS ONE style templates can be found at

https://journals.plos.org/plosone/s/file?id=wjVg/PLOSOne_formatting_sample_main_body.pdf and

https://journals.plos.org/plosone/s/file?id=ba62/PLOSOne_formatting_sample_title_authors_affiliations.pdf

Reply: the manuscript now follows PLOS ONE's style requirements.

2. One of the noted authors is a group or consortium [Sudan Analytics Research Group]. In addition to naming the author group, please list the individual authors and affiliations within this group in the acknowledgments section of your manuscript. Please also indicate clearly a lead author for this group along with a contact email address.

Reply: The affiliation of authors is Sudan Analytics Research Group, Khartoum, Sudan. The leading author is: Elfatih A. Hasabo (elfatih.ahmed.hasabo@gmail.com).

3. We note that you have indicated that data from this study are available upon request. PLOS only allows data to be available upon request if there are legal or ethical restrictions on sharing data publicly. For more information on unacceptable data access restrictions, please see http://journals.plos.org/plosone/s/data-availability#loc-unacceptable-data-access-restrictions.

Reply: The data was uploaded as a supplementary file.

In your revised cover letter, please address the following prompts:

4. We note that [Figure 1] in your submission contain [map/satellite] images which may be copyrighted. All PLOS content is published under the Creative Commons Attribution License (CC BY 4.0), which means that the manuscript, images, and Supporting Information files will be freely available online, and any third party is permitted to access, download, copy, distribute, and use these materials in any way, even commercially, with proper attribution. For these reasons, we cannot publish previously copyrighted maps or satellite images created using proprietary data, such as Google software (Google Maps, Street View, and Earth). For more information, see our copyright guidelines: http://journals.plos.org/plosone/s/licenses-and-copyright.

Reply: we deleted figure 1 and replaced with text in the manuscript.

**Reviewer 1**

We would like to thank reviewer 1 for all his hard efforts and valuable input on our manuscript.

**Specific Responses:**

***Comment 1***: “General comments

Authors are highly commended for conceptualizing this study and going ahead to conduct and also present their findings for publication. I must say that the study is of immense relevance and valuable.

While I commend the Authors for their great efforts, I am also of the opinion that some aspects of the manuscript should be revised before the manuscript could be accepted for publication.

Authors should take note of the following observations.

1. The manuscript should be reviewed critically for minor errors of grammar. Authors should delete the comment on PUBMED as an infamous database since it is a mistake. In line 142 under Introduction, Authors used ‘our study’..”

**Reply 1***:* It was corrected in the text.

***Comment 2****: “2.* Description of the outcome variable. The main outcome variable for the study included knowledge and practice of systematic review. While knowledge of systematic review was defined, practice was not. In as much, I will advise the Authors to use ≥50% of correct responses to the 19 variables used to determine knowledge as cut off for determining good knowledge of systematic review. In some instances Authors introduced secondary research in the manuscript and this appeared to run parallel to the main outcome variables. This should be clarified.”

***Reply 2****:* It was corrected in the text. The correct term systematic review.

***Comment 3****: “3.* The outcome variables should be categorized into good and poor knowledge of systematic review and good and poor practice of secondary research.

***Reply 3****: It was corrected. The secondary outcome is practicing or enrolling a secondary research which is assessed using one question .*

***Comment 4****: “4.* With such a large sample size as included in this study, Authors should endeavor to include binary logistic regression analysis in this study. This should be applied for determining the predictors of good knowledge and good practice of systematic review among the respondents. This aspect of the result should be included in the abstract..”

***Reply 4****: Logistic regression is applied only to* of good knowledge of systematic review. This is the primary outcome of the research. The second outcome which is enrolling or practicing in a secondary research is assessed using one question and it’s a secondary outcome in the research.

***Comment 5****: “*5. The presentation of the results should be reviewed to enhance the understanding of readers. For example, there is no presentation of the proportion of respondents that had good knowledge and good practice of systematic review.”

***Reply 5****:* the proportion of respondents that had good knowledge was added in the text. And percentage of respondents that had is enrolling or practicing in a secondary research was added in the text.

***Comment 6*** “*6. Authors should be mindful of use of capital letters.”*

***Reply 6****: This was corrected throughout the text.*

***Comment 7****: “*7. Authors should make statements inside the tables and avoid the use of question marks.”

***Reply 7****: we used* an independent t-test for with quantitative variables such as means. It was corrected in the text.

***Comment 8****: “*8 Other comments

Authors should consider this minor revision of the study title:

Knowledge, practice and preventive barriers towards conducting systematic reviews among undergraduate medical students of Arab countries: A multi country online survey

Abstract

Background

Replace of with among..

Methods

Knowledge about systematic reviews was assessed using 19 variables

Instead of using numbers, use proportions to determine good knowledge like ≥50% of correct responses.

Nothing was said about practice.

Results

Use majority instead of most

Why were factors affecting knowledge and practice not included?

Participants

Why was the age limitation necessary?

Revise the exclusion criteria as one has to be included before the individual could be excluded.

Use study instrument instead of instruments used to measure the variables of interest in the study.

Be specific on number of variables used to assess practice of systematic review and how the outcome variable was determined.

Statistical analysis

Use frequencies and proportions for categorical variables

Results

Participants’ characteristics

The proportion, 53% cannot be regarded as nearly half. This should be reviewed.

Discussion

Revise the phrase, infamous database……

The conclusion should be in one paragraph.”

***Reply 8****:* Title of the study has been changed into Knowledge and preventive barriers towards conducting systematic reviews among undergraduate medical students of Arab countries: A multi country online survey.

Age limitation to include all adults’ participants in this study.

Abstract background, methods, results, discussion and conclusion were edited according to the amendments.

**Reviewer 2**

We would like to thank reviewer 2 for all his hard efforts and valuable input on our manuscript.

**Specific Responses:**

***Comment 1***: “This is very important paper with new idea especially for medical research

Suggestions to improve the manuscript are as follows:

Required to address the questions:

1. What was the response rate

2. How to manage missing cases because as being online survey it was expected to have missing cases

3. This was multi country study how to ensure data safety and security”

***Reply 1****:*

1. The response rate is 89.7%.
2. All question in the online survey is obligatory; therefore there is no missing data
3. Before distribution of the survey, the collaborators invited the participants to participate in the study. Then the questionnaire was distributed to each participant by the collaborator.

***Comment 2****:*

*Suggested to add another two section*

*1.* *Data management for quality assurance and*

*2.* *Survey administration*

***Reply 2****: They were added in the methodology section.*

***Comment 3****:*

Recommendation
1. Questionnaire could be attached as a supplementary

***Reply 3****:* Thank you for the recommendation. The questions been asked in this study are found in the tables of manuscript.

Sincerely,

Elfatih A. Hasabo
