# Supplementary material for: Knowledge and preventive barriers towards conducting systematic review among undergraduate medical students of Arab countries: A multi country online survey
Source: PLoS One. 2025 Aug 18;20(8):e0329827. doi: 10.1371/journal.pone.0329827 (PMC12360549; doi:10.1371/journal.pone.0329827)
Supplement: Attachment — Submitted filename: Review_comments_declet_1.docx [file pone.0329827.s006.docx]

**Review comments**

**General comments**

I thank the Editor for the opportunity to review this manuscript. I also commend the Authors for the good review of the manuscript. To a large extent, there are still some aspects of the manuscript that may require a further revision

Authors should take note of the following observations:

1. Authors should review the manuscript critically for minor errors of grammar.
2. Objectives of the study. The objectives of the study as expressed in the abstract did not include the aspect concerning secondary research.
3. The calculation of the response rate has brought forward some unanswered questions. For example, how many countries were included in the study? What is the number of medical schools in the countries including private and public universities and the number of medical students? The basis for the calculation of the response rate should be explained. It may be important to include the steps taken by the Authors in ensuring that the questionnaire was completed only by those who were eligible. The number of submitted questionnaires which were not included in the analysis because of incomplete information should also be stated.
4. It may be good to indicate how the differences in the exposure of the medical students to research and also systematic review in the different countries affected the outcome of the study. This may require that an insight into the medical curriculum of those countries included in the study are provided. Equally important is the role of members of the Faculty. If the Lecturers provided support to the student, is it because it is part of their responsibilities or that they are including the medical students in their own personal research programs. For example, the students that have published, did they do that alone or in conjunction with their Lecturers.
5. Presentation of the results. The presentation of the results of the study is not clear. There is no table of figure depicting the main outcome measure of the study. I will prefer that the socio-demographic characteristics of the respondents (alone) be presented as table 1. This is important bearing in mind that this is a multi -country study. Table 2 should focus on the knowledge of systematic review. What is the purpose of table 2 as presented and how was the 2x2 Chi square table generated? Why was it necessary to use column percent when the outcome variable was at the column section? Again, it is better to make statement in tables and not ask questions. In table 3, Authors should use proportions to present Chi square test instead of odds ratio. Why was it necessary to include the respondents by year of study and also current level in medical faculty. Authors should use adjusted odds ratio for multivariate logistic regression instead of odds ratio. Why was marital status and other variables not included in the regression model? Why was age not categorized? What is the basis for inclusion of variables into the logistic regression model after bivariate Chi square analysis? Why were the variables in table 4 cross-tabulated with the outcome variable? There are no explanations in the manuscript on how table 5 was derived. Arrange the responses in table 6 in descending order.
6. Abstract. It will be better to state that 19 variables were used to assess knowledge of systematic review and that respondents that correctly answered ≥50% of the variables were regarded as having good knowledge. Then in the methodology section of the manuscript, it is important to state that a correct response by any respondent attracted a score of one while an incorrect answer was scored zero. Do not start a sentence with a figure. Include the response rate in the abstract. Use adjusted odds ratio (AOR) and delete p values. Regarding practice ….. (of what). Was the focus of the study centered on the students publishing or specifically publishing secondary research?
7. Revise the last sentence in the Introduction section. Also, lines 217 -219.
8. Is it pre-testing of study tools that was done or pilot testing? This should be clarified.
9. Calculation of sample size. This study is a total population study. Why was it necessary to calculate sample size. The sample size calculation should be deleted.
10. In lines 257 and 258, the respondents should sign a written informed consent form and not ‘provide.’ How did the respondents ‘provide’ a clear explanation of the study purpose.
11. Categorical variables were summarized using frequencies and proportions.
12. Use highest proportion of the students, 32.1% were from Suan instead of ‘most’.
13. Discussion. Delete the confidence intervals and p values in the discussion.
