# Supplementary material for: Knowledge and preventive barriers towards conducting systematic review among undergraduate medical students of Arab countries: A multi country online survey
Source: PLoS One. 2025 Aug 18;20(8):e0329827. doi: 10.1371/journal.pone.0329827 (PMC12360549; doi:10.1371/journal.pone.0329827)
Supplement: Attachment — Submitted filename: Point_to_point_response_R2_PLOS.docx [file pone.0329827.s007.docx]

**Point-by-point responses**

**Title of Article:** Knowledge, practice, and perceived barriers towards conducting systematic reviews in undergraduate medical students of Arab countries: A multi-country online survey

**Name of the Corresponding Author:** Elfatih A. Hasabo

**Email Address of the Corresponding Author:** elfatih.ahmed.hasabo@gmail.com

**Reviewer 1**

We thank reviewer 1 for all his hard efforts and valuable input on our manuscript.

**Specific Responses:**

***Comment 1***: *Authors should review the manuscript critically for minor errors of grammar.*

**Reply 1**: The authors revised the manuscript thoroughly for grammatical errors. A Grammarly check was also performed.

***Comment 2****: Objectives of the study. The study's objectives, as expressed in the abstract, did not include the aspect concerning secondary research.*

**Reply 2**: We clarified the study aim in the abstract. Our study addressed three major aspects: the students' knowledge, practices, and barriers to conducting secondary research.

***Comment 3****: The calculation of the response rate has brought forward some unanswered questions. For example, how many countries were included in the study? What is the number of medical schools in the countries including private and public universities and the number of medical students? The basis for the calculation of the response rate should be explained. It may be important to include the steps taken by the Authors in ensuring that the questionnaire was completed only by those who were eligible. The number of submitted questionnaires which were not included in the analysis because of incomplete information should also be stated.*

***Reply 3****: Thank you for your comment. There are 9 countries were included in this study, including 113 medical school. This research was conducted during COVID-19 pandemic. Therefore, the only way was to use the online survey. We send invitation for participation in this study and the participants who agreed to participate we send the link for them. Then, response rate is the percentage of student who agreed to participate in this study. As we know the access to the internet including the slow connection and availability of mobile phone may restrict some student from participating and this was mentioned in the limitation section. There is an option to make all questions mandatory and you can’t submit without writing the answered there; here we ensured everyone have the questionnaire submitted the full questionnaire.*

***Comment 4****: It may be good to indicate how the differences in the exposure of the medical students to research and systematic review in the different countries affected the study's outcome. This may require that an insight into the medical curriculum of those countries included in the study are provided. Equally important is the role of members of the Faculty. If the Lecturers supported the student, is it because it is part of their responsibilities or that they are including the medical students in their own personal research programs. For example, the students who have published, did they do that alone or in conjunction with their Lecturers?*

***Reply 4****:*

Thanks a lot sir for your time and supporting comments. Requested changes have been made.

***Comment 5****: Presentation of the results. The presentation of the results of the study is not clear. There is no table of figure depicting the main outcome measure of the study. I will prefer that the socio-demographic characteristics of the respondents (alone) be presented as table 1. This is important bearing in mind that this is a multi -country study. Table 2 should focus on the knowledge of systematic review. What is the purpose of table 2 as presented and how was the 2x2 Chi square table generated? Why was it necessary to use column percent when the outcome variable was at the column section? Again, it is better to make statement in tables and not ask questions. In table 3, Authors should use proportions to present Chi square test instead of odds ratio. Why was it necessary to include the respondents by year of study and also current level in medical faculty. Authors should use adjusted odds ratio for multivariate logistic regression instead of odds ratio. Why was marital status and other variables not included in the regression model? Why was age not categorized? What is the basis for inclusion of variables into the logistic regression model after bivariate Chi square analysis? Why were the variables in table 4 cross-tabulated with the outcome variable? There are no explanations in the manuscript on how table 5 was derived. Arrange the responses in table 6 in descending order.*

***Reply 5****:* We converted all the questions in the tables into statements and rearranged the responses in table 6 in a decending order.

***Comment 6***: *Abstract. It will be better to state that 19 variables were used to assess knowledge of systematic review and that respondents that correctly answered ≥50% of the variables were regarded as having good knowledge. Then in the methodology section of the manuscript, it is important to state that a correct response by any respondent attracted a score of one while an incorrect answer was scored zero. Do not start a sentence with a figure. Include the response rate in the abstract. Use adjusted odds ratio (AOR) and delete p values. Regarding practice ….. (of what). Was the focus of the study centered on the students publishing or specifically publishing secondary research?*

***Reply 6****:* The reviewer's comments on the abstract and methodology were addressed. We further clarified the method used in evaluating the students' level of knowledge about secondary research in the abstract and methods as suggested by the reviewer. As well, the response rate was added, the P-values ad confidence intervals were deleted, and the studied aspect of students' practices was clarified in the abstract.

***Comment 7****: Revise the last sentence in the Introduction section. Also, lines 217 -219.*

***Reply 7****:* The sentences were revised and clarified.

***Comment 8****: Is it pre-testing of study tools that was done or pilot testing? This should be clarified.*

***Reply 8****: Yes, it was done and mentioned in the study.*

***Comment 9****:* *Calculation of sample size. This study is a total population study. Why was it necessary to calculate the sample size? The sample size calculation should be deleted.*

***Reply 9****: this section has been deleted.*

***Comment 10****:* *In lines 257 and 258, the respondents should sign a written informed consent form and not ‘provide.’ How did the respondents ‘provide’ a clear explanation of the study purpose.*

***Reply 10****:* The authors corrected the statement on the informed consent. As the study questionnaire was provided via an online form, the respondents did not sign the consent. Rather, and explanation of the study purposes was initially provided in the online form and the first question in the form was about the respondents' consent to participate. Only after agreeing to participate in the study, the respondents filled the research questionnaire.

***Comment 11****:* *Categorical variables were summarized using frequencies and proportions.*

***Reply 11****:* The proportions were added to correctly complete the sentence.

***Comment 12****:* *Use highest proportion of the students, 32.1% were from Sudan instead of ‘most’.*

***Reply 12****:* The expression was corrected.

***Comment 13****:* *Discussion. Delete the confidence intervals and p values in the discussion.*

***Reply 13****:* The confidence intervals and p values were removed from the discussion.

**Reviewer 2**

We thank reviewer 2 for all his strenuous efforts and valuable input on our manuscript.

**Reply**: thank you for your valuable comments in the PDF file. All your points were addressed in the text.

**Specific Responses:**

***Comment 1****:*

*Required to explain more about the questionnaire development and scoring procedure*

*How you ensure reliability and validity of the question*

*What was the evidence for 9 is there have any reference*

***Reply****: the questionnaire was developed after extensive literature review and expert opinion because no previous study was conducted about this topic. The questionnaire underwent face validity checks, and pilot testing. The cut-off score of 9 is the 50% percentage of the questions a used in previous studies assessed the knowledge of other topics*

***Comment 2****:*

*1. What was the response rate*

*2. How to manage missing cases because as being online survey it was expected to have missing cases*

*3. This was multi country study how to ensure data safety and security*

***Reply****: response rate is 89.7%. We managed missing by adding an option that all questions are compulsatory and need to be all answered.*

***Comment 3****:*

*Suggested to add another two section*

*1. Data management and*

*2. Survey administration*

***Reply****: it was added.*

***Comments 4****:*

*How to identify top universities*

*Used any ranking criteria like QS or THE*

*If the university is research oriented (giving emphasis on research) were there have any knowledge difference*

***Reply****: Top universities was reported by a previous study which is according to QS ranking.*

**Comments 5**:

Better to represent GRAPH using statistics (percentage) instead of number

**Reply**: it was reported in the text as percentage, and no need to put the figure

**Comment 6**:

As different statistics were used so p value should be differentiate using different symbol

Reply: Yes, for continuous variables, we used Two Sample t-test and categorical we used Fisher's exact test and Chi-squared test where they mentioned in the methodology section.

**Comment 7**:

This column is unnecessary

Better to present the sample size in the title as it is equal number for all variables

**Reply**: it was deleted.

Sincerely,

Elfatih A. Hasabo
