# Supplementary material for: Knowledge and preventive barriers towards conducting systematic review among undergraduate medical students of Arab countries: A multi country online survey
Source: PLoS One. 2025 Aug 18;20(8):e0329827. doi: 10.1371/journal.pone.0329827 (PMC12360549; doi:10.1371/journal.pone.0329827)
Supplement: Attachment — Submitted filename: Review_comments_declet_2.docx [file pone.0329827.s008.docx]

**Review comments**

**General comments**

I commend the Authors for the good work and the good review of the manuscript. That notwithstanding, I am of the opinion that certain aspects of the manuscript may require a further revision before the manuscript could be accepted for publication.

Authors should take note of the following observations:

1. Sample size determination. Authors indicated in the abstract that this was a total population study and included the response rate. However, in the methods section of the manuscript retained the sub-section on sample size calculation. This should be deleted and a description of total population study and the response rate included.
2. Authors should use adjusted odds ratio for multivariate analysis instead of Odds ratio, (OR). If adjusted odds ratio is reported in the abstract, there may be no need to include the p value so the p value in the abstract should be deleted.
3. Authors should bear in mind the title of the study. The last sentence of the Introduction section defined another major objective of the study which is at variance with the title of the study. This should be reconciled.
4. Table 3 is a bit confusing especially when one considers the results of year of study under univariate logistic regression analysis. Authors should present Chi square analysis instead of univariate logistic regression analysis clearly indicating the proportions as obtained from the analysis. Authors should use adjusted odds ratio instead of odds ratio for reporting the results of multivariate logistic regression analysis. **It is important for the Authors to indicate the basis for inclusion of variables into the logistic regression model after Chi square test analysis.**
5. In table 1, The column Yes for ‘Enrolled or practiced in secondary research’ should come before the column, No.
6. Odds ratio as included in the discussion section should be deleted.
7. Line 124 in the abstract section should be reviewed.
