# Supplementary material for: Knowledge and preventive barriers towards conducting systematic review among undergraduate medical students of Arab countries: A multi country online survey
Source: PLoS One. 2025 Aug 18;20(8):e0329827. doi: 10.1371/journal.pone.0329827 (PMC12360549; doi:10.1371/journal.pone.0329827)
Supplement: Attachment — Submitted filename: Point_to_point_response_R2_PLOS_auresp_3.docx [file pone.0329827.s009.docx]

**Point-by-point responses**

**Title of Article:** Knowledge, practice, and perceived barriers towards conducting systematic reviews in undergraduate medical students of Arab countries: A multi-country online survey

**Name of the Corresponding Author:** Elfatih A. Hasabo

**Email Address of the Corresponding Author:** elfatih.ahmed.hasabo@gmail.com

**Reviewer 1**

We thank reviewer 1 for all his hard efforts and valuable input on our manuscript.

**Specific Responses:**

***Comment*** :

I commend the Authors for the good work and the good review of the manuscript. That notwithstanding, I am of the opinion that certain aspects of the manuscript may require a further revision before the manuscript could be accepted for publication.

Authors should take note of the following observations:
1. Sample size determination. Authors indicated in the abstract that this was a total population study and included the response rate. However, in the methods section of the manuscript retained the sub-section on sample size calculation. This should be deleted and a description of total population study and the response rate included.

**Reply**:

The sample size calculation section has been deleted.

***Comment***:

2. Authors should use adjusted odds ratio for multivariate analysis instead of Odds ratio, (OR). If adjusted odds ratio is reported in the abstract, there may be no need to include the p value so the p value in the abstract should be deleted.

**Reply**:

P values has been deleted from the abstract

***Comment***:
3. Authors should bear in mind the title of the study. The last sentence of the Introduction section defined another major objective of the study which is at variance with the title of the study. This should be reconciled.

**Reply**:

Thank you for your comment. It has been in the text.

***Comment***:
4. Table 3 is a bit confusing especially when one considers the results of year of study under univariate logistic regression analysis. Authors should present Chi square analysis instead of univariate logistic regression analysis clearly indicating the proportions as obtained from the analysis. Authors should use adjusted odds ratio instead of odds ratio for reporting the results of multivariate logistic regression analysis. It is important for the Authors to indicate the basis for the inclusion of variables into the logistic regression model after the chi-square test analysis.
**Reply**:

Thank you for your comment. We deleted the univariate logistic regression analysis from Table 3. The values of the chi-square test were already presented in Table 1. Moreover, adjusted odds ratio has been used for reporting the results of multivariate logistic regression, and variables with a p-value <0.05 were entered in the multivariate logistic regression.

***Comment***:

5. In table 1, The column Yes for ‘Enrolled or practiced in secondary research’ should come before the column, No.
**Reply**:

Thank you for your comment is has been corrected.

***Comment***:

6. Odds ratio as included in the discussion section should be deleted.
**Reply**:

The odds ratio has been deleted

***Comment***:

7. Line 124 in the abstract section should be reviewed.

**Reply**:

it has been corrected

**Reviewer 2**

We thank reviewer 2 for all his strenuous efforts and valuable input on our manuscript.

**Reply**: thank you for your valuable comments in the PDF file. All your points were addressed in the text.

**Specific Responses:**

***Comment****:*

*1. Reliability coefficient value (for example, Cronbach's alpha) should be mentioned for different sections of the questionnaire like: Knowledge, practice, and* *perceived barriers*

***Reply****:*

*Thank you for your comment.* *The Cronbach's alpha was measured and was found to be 0.861 for* *knowledge, 0.879 for perceived barrier, 0.963 for Self-rated skills, and 0.95 for* *practice. They were reported in the text.*

***Comment****:*

*2. Better to mention the response rate in the manuscript. Still not addressed the comments on data safety and security (how maintained) issues.*

***Reply****:*

The response rate has been mentioned in the text in the results section, and it's 89.7%.

Sincerely,

Elfatih A. Hasabo
